# Step-by-Step Modulation of Crystalline Features and Exciton Kinetics for 19.2% Efficiency Ortho-Xylene Processed Organic Solar Cells

**DOI:** 10.1007/s40820-023-01241-z

**Published:** 2023-11-23

**Authors:** Bosen Zou, Weiwei Wu, Top Archie Dela Peña, Ruijie Ma, Yongmin Luo, Yulong Hai, Xiyun Xie, Mingjie Li, Zhenghui Luo, Jiaying Wu, Chuluo Yang, Gang Li, He Yan

**Affiliations:** 1 Department of Chemistry Department of Chemistry and Hong Kong Branch of Chinese National Engineering Research Center for Tissue Restoration and Reconstruction, The Hong Kong University of Science and Technology, Clear Water Bay, Hong Kong, People’s Republic of China; 2 Department of Electronic and Information Engineering, Research Institute for Smart Energy (RISE), Guangdong-Hong Kong-Macao (GHM) Joint Laboratory for Photonic-Thermal-Electrical Energy Materials and Devices, The Hong Kong Polytechnic University, Hung Hom, Kowloon, Hong Kong, 999077 People’s Republic of China; 3 The Hong Kong University of Science and Technology, Function Hub, Advanced Materials Thrust, NanshaGuangzhou, 511400 People’s Republic of China; 4 Department of Applied Physics, The Hong Kong Polytechnic University, Hong Kong, People’s Republic of China; 5 Shenzhen Key Laboratory of New Information Display and Storage Materials, College of Materials Science and Engineering, Shenzhen University, Shenzhen, 518060 People’s Republic of China

**Keywords:** Organic solar cells, Ternary design, Solvent selection, Flouro-methoxylated end group, Morphological ordering

## Abstract

With plenty of popular and effective ternary organic solar cells (OSCs) construction strategies proposed and applied, its power conversion efficiencies (PCEs) have come to a new level of over 19% in single-junction devices. However, previous studies are heavily based in chloroform (CF) leaving behind substantial knowledge deficiencies in understanding the influence of solvent choice when introducing a third component. Herein, we present a case where a newly designed asymmetric small molecular acceptor using fluoro-methoxylated end-group modification strategy, named BTP-BO-3FO with enlarged bandgap, brings different morphological evolution and performance improvement effect on host system PM6:BTP-eC9, processed by CF and ortho-xylene (*o*-XY). With detailed analyses supported by a series of experiments, the best PCE of 19.24% for green solvent-processed OSCs is found to be a fruit of finely tuned crystalline ordering and general aggregation motif, which furthermore nourishes a favorable charge generation and recombination behavior. Likewise, over 19% PCE can be achieved by replacing spin-coating with blade coating for active layer deposition. This work focuses on understanding the commonly met yet frequently ignored issues when building ternary blends to demonstrate cutting-edge device performance, hence, will be instructive to other ternary OSC works in the future.

**Supplementary Information:**

The online version contains supplementary material available at 10.1007/s40820-023-01241-z.

## Highlights

- A novel fluoro-methoxylated end group for Y-series acceptors is produced, and asymmetric substitution strategy is applied as a step-by-step optimization.
- 19.24% power conversion efficiency is achieved for industrially compatible solvent ortho-xylene processed organic solar cells.
- Underlying morphological and photo-physical variation is revealed for device performance difference brought by solvent selection, which could set up a template for future research on similar topics.

**Supplementary Information:**

The online version contains supplementary material available at 10.1007/s40820-023-01241-z.

## Graphical abstract

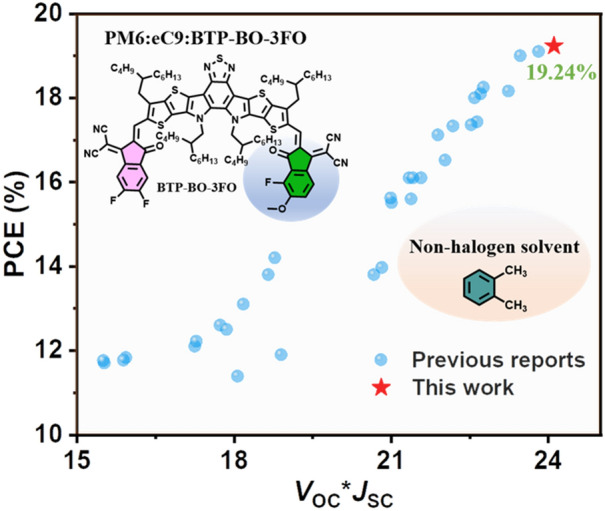

## Introduction

Organic solar cells (OSCs), that are of great promise in carbon-zero society and smart city construction as energy supplier, have achieved over 19% power conversion efficiency (PCE) in single-junction devices, and > 20% values in tandem structures [1–16]. This is mainly attributed to rational material design and combination, yet also thanks to chloroform (CF), a powerful solvent (solubility) enabling quick liquid-to-solid phase transition, which makes thin-film morphology tuning easier than high boiling point (BP) solvent-based systems [5, 17–23]. However, CF is not an ideal choice for mass production of OSC devices, especially in printing-type scenarios, whose low BP would make the film formation process hard to control. Besides, its potential carcinogenicity is an evitable disadvantage. Thereby, realizing high PCE in high BP, non-halogenated solvents, such as ortho-xylene (industrially compatible)-processed OSCs, is of great significance.

Designing novel materials with appreciable features, e.g., desired energy level, strong crystallinity, and good solubility, as a third component to construct ternary blend with host donor–acceptor system has been a popular and facile strategy to improve the device efficiency in recent years [24–33]. Notably, almost all reports focus on CF-processed device to evaluate material performances, for that a basic hypothesis is other solvents enabled devices shall demonstrate similar efficiency variation tendency. Therefore, it is still a blank in the research field of reporting solvent selection induced ternary device working mechanism difference: Rare cases focus on whether processing solvent has impact on the sucess of morphology optimization when ternary strategy is applied.

**Fig. 1:**
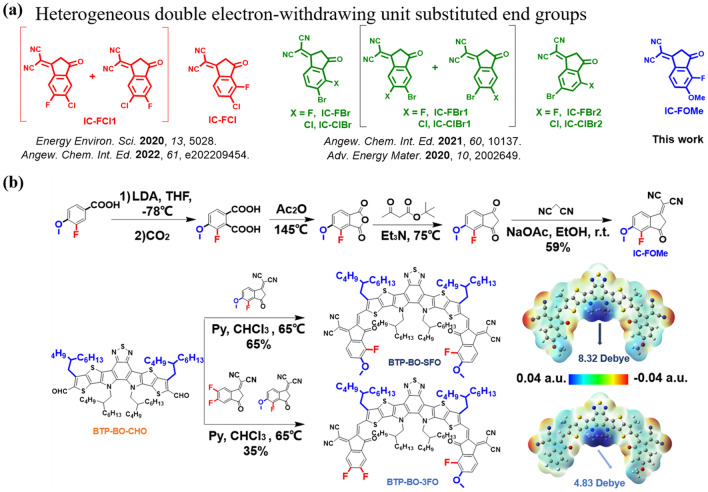
**a** Design strategy of IC-FOMe; **b** Synthetic route and electrostatic potential of BTP-BO-3FO and BTP-BO-SFO

On the other hand, the development of small-molecule acceptors (SMAs) with high lowest unoccupied molecular orbital (LUMO) energy levels as the third component is an important strategy to enhance the efficiency of ternary devices. The terminal group, as an essential “A” component of A − DA′ D − A-type NFAs, plays non-negligible roles in modulating the absorption spectrum, determining LUMO level and intermolecular charge transfer (ICT) effect. Currently, for ADA_1_DA-type SMAs, halogenated substituted terminal groups have shown great advantages in developing acceptors with large dipole moments, strong crystallinity, and high device performance. Besides, the position of the halogen in the hetero-di-halogenated substituted terminal group also exerts a notable influence on the physicochemical properties of the molecule. For instance, we have developed three isomeric Cl/Br co-substituted terminal groups (IC-ClBr with *β*- and *γ*-halogen substitution sites, IC-ClBr1 with *γ*- and *δ*-halogen substitution sites, and IC-ClBr2 with *β*- and *γ*-halogen substitution sites) by changing the position of Cl and Br on the terminal benzene (Fig. 1a) [34]. Among them, the IC-ClBr-terminated acceptor-based device demonstrated higher open-circuit voltage (*V*_OC_) than that of IC-ClBr1- and IC-ClBr2-terminated acceptor (0.906 vs. 0.854 vs. 0.845 V). Similarly, the fluorine-chloride co-substituted terminal groups reported by Wang and co-workers also showed the same trend of *V*_OC_ change. Furthermore, the incorporation of electron-donating units (methyl, methoxyl) into terminal group could effectively upshift LUMO value and reduce the optical bandgap, thereby enhancing the *V*_OC_ of OSCs. Hence, the development of terminal groups with specific positions of a halogen and an electron-donating unit presents a promising avenue for creating acceptors with high *V*_OC_ and good efficiency [35]. Additionally, there have been limited reports on terminal groups that combine both electron-donating and electron-withdrawing units. Exploring such combinations will not only expand the range of available terminal groups but also enrich the material library for both terminal groups and SMAs.

Drawing upon these points, we have synthesized a fluorine- and methoxy-co-substituted terminal group with *β*- and *γ*-substitution sites and successfully incorporated them into SMAs, which leads to the creation of two different acceptors, namely symmetric BTP-BO-SFO and asymmetric BTP-BO-3FO. Both materials contain enlarged bandgaps and yield high *V*_OC_ in binary devices matching with the celebrity polymer donor PM6. Though higher *V*_OC_ of 1.00 V is achieved by BTP-BO-SFO-based cell, its poor short circuit current density (*J*_SC_) and fill factor (*FF*) leads to a low PCE of 10.2%, thus not an ideal material for ternary blend construction. In contrast, the asymmetric BTP-BO-3FO realizes a decent efficiency as high as 15.80%, while the *V*_OC_ still locates at a satisfactory level of 0.952 V. Then, BTP-BO-3FO is incorporated into a representative high-efficiency (in both CF- and o-XY-treated devices) binary system PM6:BTP-eC9 to pursue a greater photovoltaic performance. As a result, CF-processed ternary cells with an optimized ratio produces a 18.60% efficiency compared to 18.29% of binary counterpart. Although enhanced *V*_OC_ and* J*_SC_, decreased *FF* limits the general PCE improvement. Meanwhile, *o*-XY-processed cells display a more significant rise in PCE due to simultaneously improved *V*_OC_,* J*_SC_, and *FF*. A series of morphological and photo-physics characterizations reveal such different behaviors are caused by different effect upon film morphology, i.e., CF-processed ternary films show much more loss in order phase proportion and crystalline ordering, while *o*-XY cast series demonstrate less π–π stacking ordering and general order phase (desired electronic transition). This difference is further confirmed by charge behavior characterization, from which we can learn that *o*-XY-processed ternary film’s charge transfer and recombination kinetics is more favorable. This study not only realizes a cutting-edge efficiency value for application favorable *o*-XY-processed OSCs, but also provides a new case and understanding where ternary blend construction combined suitable solvent selection can maximize the expected improvement.

## Experimental Section

### Materials

PBDB-T-2F, PBDB-TF or PM6: poly[(2,6-(4,8-bis(5-(2-ethylhexyl-3-fluoro)thiophen-2-yl)-benzo[1,2-b:4,5-b′]dithiophene))-alt-(5,5-(1′,3′-di-2-thienyl-5′,7′-bis(2-ethylhexyl)benzo[1′,2′-c:4′,5′-c′]dithiophene-4,8-dione)].

BTP-eC9: 2,2′- [[12,13-Bis(2-butyloctyl)-12,13-dihydro-3,9-dinonylbisthieno[2″,3″:4′,5′]thieno[2′,3′:4,5]pyrrolo[3,2-e:2′,3′-g][2,1,3]benzothiadiazole-2,10-diyl]bis[methylidyne(5,6-chloro-3-oxo-1H-indene-2,1(3H)-diylidene)]]bis[propanedinitrile].

PFN-Br: Poly(9,9-bis(3′-(N,N-dimethyl)-N-ethylammoinium-propyl-2,7-fluorene)-alt-2,7-(9,9-dioctylfluorene))dibromide.

All above materials are purchased from Solarmer Inc.

MA: melamine.

PFN-Br-MA: doped PFN-Br by MA.

Chloroform, chlorobenzene and ortho-xylene are from Sigma-Aldrich Inc.

PEDOT:PSS with the type of Clevios P VP AI 4083 was obtain from Heraeus.

The leaser patterned ITO substrates (15 Ω sq^−1^) were purchased from were obtained from South China Xiang City Inc.

All reagents and solids were used as received without any further purification.

### Device Fabrication and Characterization

Solar cells were fabricated in a conventional device configuration of ITO/PEDOT:PSS-TA/active layers/PFN-Br-MA/Ag. The ITO substrates (~ 94% transmittance) were first scrubbed by detergent and then sonicated with deionized water, acetone and isopropanol subsequently, and dried overnight in an oven. The glass substrates were treated by UV ozone for 30 min before use. PEDOT:PSS-TA (1 mg mL^−1^ tyramine doped in standard Hareus Al 4083 solution) was spin-cast onto the ITO substrates at 6000 rpm for 30 s, and then dried at 160 °C for 15 min in air. The blend of PM6:acceptors (eC9, BTP-BO-3FO, BTP-BO-SFO, and derived alloys) (1:1.3 in weight) were dissolved in CF (7 mg mL^−1^ donor concentration) and o-XY (10 mg mL^−1^ donor concentration), with DIO (0.3% vol in CF and 0.5% vol in o-XY, respectively) as additive, and stirred on a 40 °C (for CF)/100 °C (for o-XY) hotplate for 20 min in a nitrogen-filled glove box. The blend solution was spin-cast at 2500 rpm for 30 s onto PEDOT:PSS-TA films followed by a temperature anealing of 100 °C for 1 min. PFN-Br-MA (melamine doped with 0.25% weight ratio) thin layers were coated on the active layer with 3000 rpm (0.5 mg mL^−1^), followed by the deposition of Ag (100 nm) (evaporated under 1 × 10^–3^ Pa through a shadow mask). The optimal active layer thickness measured by a Bruker Dektak XT stylus profilometer was 100–110 nm. The current density–voltage (*J–V*) curves of devices were measured using a Keysight B2901A Source Meter in glove box under AM 1.5G (100 mW cm^−2^) using a Enlitech solar simulator. The device contact area was 0.05 cm^2^, device illuminated area during testing was 0.04 cm^2^, which was determined by a mask. The EQE spectra were measured using a Solar Cell Spectral Response Measurement System QE-R3011 (Enlitech Co., Ltd.). The light intensity at each wavelength was calibrated using a standard monocrystalline Si photovoltaic cell.

### Open-air Blade Coating for Active Layers

The blade coat films were fabricated by o-XY solutions (11 mg mL^−1^ donor concentration, for binary and ternary blends; 100 °C to ensure materials are fully dissolved) with a 35 mm s^−1^ speed forward and backward (the blade-substrate gap is c.a. 120 μm) on room temperature ITO/PEDOT:PSS substrates and then transferred (after c.a. 15 s) to a nearby 100 °C hotplate to be annealed for 1 min. These steps are all carried out in ambient atmosphere with a 70% RH humidity. The N_2_ knife is used to properly accelerating the film drying, to make the blade-coated film has a similar drying kinetics as spin-coated counterparts does, and prevent any unexpected morphology destruction caused by too slow evaporation. The knife is achieved by gas pipeline releasing N_2_ with controllable intensity and direction (normally parallel to the film).

## Results and Discussion

### Material Eigen Properties

Figure 1b presents the detailed synthetic routes to IC-FOMe and two SMAs, BTP-BO-SFO and BTP-BO-3FO via a series of mature reactions reported before. The synthesis procedures and characterization data, including NMR and mass spectra, are provided in Figs. S1–S7. Note the end group IC-FOMe shall have two isomers called IC-FOMe-A and IC-FOMe-B, as demonstrated in Fig. S1. They are not likely to be separated due to highly similar polarities. Fortunately, based on low yield of IC-FOMe-B and further NMR results, we can confirm the terminal group on BTP-BO-3FO is IC-FOMe-A. The thermal stability and thermodynamics of BTP-BO-SFO and BTP-BO-3FO were investigated through thermogravimetric analysis (TGA) and differential scanning calorimetry (DSC) measurement, as shown in Fig. S8. The results indicated that both BTP-BO-SFO and BTP-BO-3FO possess great thermal stability with decomposition temperature (T_d_, 5% weight loss) over 300 °C. Furthermore, the melting point of BTP-BO-3FO locates at higher temperature than BTP-BO-SFO according to the DSC curves, suggesting superior molecular packing and stronger crystallinity of BTP-BO-3FO compared to BTP-BO-SFO. Subsequently, cyclic voltammetry was carried out to investigate the energy alignment of those two new acceptors with the credited donor PM6 and the results are shown in Fig. S9. The lowest unoccupied molecular orbital (LUMO)/highest occupied molecular orbital (HOMO) levels were determined to be − 3.30/− 5.53 and − 3.52/− 5.60 eV for BTP-BO-SFO and BTP-BO-3FO, respectively. Besides, density functional theory (DFT) calculations based on the wb97xd/6–31 + g(d,p) level were conducted to investigate the electrostatic potential (ESP) and molecular dipole moment (Figs. 1b and S10). Both new acceptors possess positive electrostatic potential (ESP) and high dipole moment, which could facilitate charge separation and self-assembly of molecules for ordered molecular stacking. Focusing on the end groups in the BTP-BO-3FO and BTP-BO-SFO, we observed that the incorporation of methoxy groups leads to a reduction in the ESP of the benzene due to the electron-donating proper of the methoxy groups (from 0.0194 to 0.0117 a.u. in Fig. S11), resulting in increased electron density and diminished intramolecular ICT effects. Additionally, BTP-BO-3FO shows smaller dipole moment of 4.83 Debye than that of BTP-BO-SFO (8.32 Debye), which could be ascribed to the lower ESP of benzene ring in IC-FO relative to that of the benzene ring in IC-2F [36].

Subsequently the photovoltaic performances of these two materials are evaluated by fabricating a series of devices based on traditional configuration of ITO/PEDOT:PSS-TA/active layer/PFN-Br-MA/Ag [37, 38]. The current density vs voltage (*J-V*) characteristics are drawn in Fig. S12a, wherein specific parameters (*V*_OC_,* J*_SC_, *FF* and PCE) are also notated. As a result, The PM6:BTP-BO-SFO blend yields an efficiency of 10.23%, due to its poor *J*_SC_ and *FF*, which indicates though high *V*_OC_, its interplay with binary host materials is supposed to be harmful to charge generation/recombination dynamics. On the contrary, BTP-BO-3FO can realize a 15.80% PCE in binary devices, where *J*_SC_ and *FF* values are decent. Therefore, this material, thanks to the finely tuned properties by new end-group design and asymmetric substitution strategy, becomes promising in the high-efficiency ternary blend construction. In addition, the reliability of testing results is confirmed by the external quantum efficiency (EQE) spectra and integrated current density values, as shown in Fig. S12b.

### Photovoltaic Performance

**Fig. 2:**
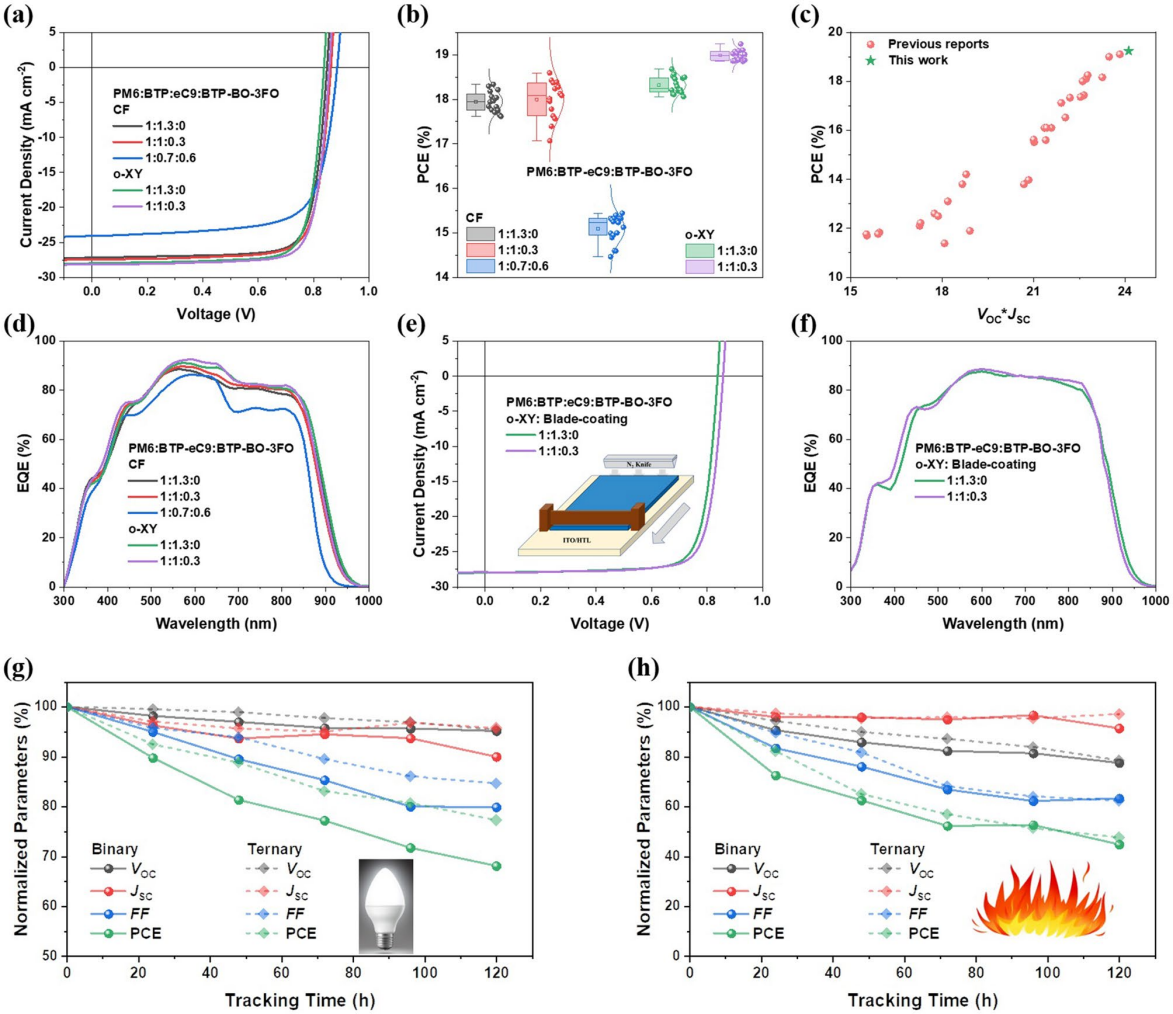
**a** *J-V* characteristics. **b** Normal distribution of PCEs. **c** A comparison of non-halogenated solvent processed OSCs. **d** EQE spectra. Blade-coating active layer from o-XY based binary and ternary solar cell performances: **e** *J-V* curves and **f** EQE spectra. **g** Light soaking stability of o-XY fabricated devices, and corresponding **h** thermal stability (80 °C)

Next, the ternary blend construction is carried out by combining BTP-BO-3FO with PM6:BTP-eC9 host system, whose CF- and o-XY-processed devices both performs well. Corresponding *J-V* curves, extracted parameters, and their normal distribution plots are given in Figs. 2 and S13, Table 1. Within CF-processed cells, the component optimization is also carried out: 1:1:0.3 weight ratio for PM6:BTP-eC9:BTP-BO-3FO is the best. Both *V*_OC_ and* J*_SC_ are improved there, but *FF* suffers some loss, which leads to insufficient efficiency promotion. When BTP-BO-3FO’s content becomes larger, the PCE drops quickly to 15.42%, even lower than its own binary devices. This variation indicates that BTP-BO-3FO is capable of tuning energy level and optimize absorption spectra, but has negative impact on film morphology, under CF casting condition. On the contrary, *o*-XY-processed 1:1:0.3 devices have a more significantly improved PCE than its counterpart, thanks to simultaneously enhanced *V*_OC_,* J*_SC_, and *FF*, which implies a different morphology evolution in *o*-XY cast active layers. Notable 19.24% is among the highest level of non-halogenated solvent processed OSC efficiencies, which is then emphasized by a brief comparison shown as Fig. 2c. The literature values are listed in Table S1. Furthermore, EQE spectra are measured and presented as well, in Fig. 2d. Since o-XY’s greener nature and high boiling point, our research interest drives us to further explore the ternary strategy’s effect on blade-coating (a printing compatible fabrication) cells. The results are shown in Fig. 2e, f and Table 1. Over 19% efficiency for non spin-coating devices based on easy-controllable solvent is for the first time achieved.

**Table 1:** Device performances

PM6:BTP-eC9:BTP-BO-3FO	*V*_OC_ (V)	*J*_SC_ /* J*_EQE_ (mA cm^−2^)	*FF* (%)	PCE (%)
*CF processed*				
1:1.3:0	0.849	27.17/26.25	79.3	18.29 (17.95 ± 0.23)
1:1:0.3	0.863	27.42/26.40	78.6	18.60 (17.99 ± 0.45)
1:0.7:0.6	0.885	24.07/23.66	72.4	15.42 (15.09 ± 0.30)
*o-XY processed*				
1:1.3:0	0.838	27.97/27.09	79.5	18.63 (18.32 ± 0.19)
1:1:0.3	0.857	28.13/27.16	79.8	19.24 (18.99 ± 0.12)
*o-XY blade coating*				
1:1.3:0	0.840	27.59/26.60	79.5	18.42
1:1:0.3	0.857	27.73/26.73	79.7	19.05

The brackets contain averages and standard errors of PCEs based on at least 10 devices

Beyond efficiency, the light-soaking stability and thermal stability of encapsulated devices are paid attention for non-halogenated solvent processed groups, as well. According to Fig. 2g, h, where the results are plotted, the addition of BTP-BO-3FO is beneficial to prolonging the lifetime for those under white LED illumination under ambient condition, while long-term thermal stability varies little when they are on 80 °C hotplate for thermal degradation, which is supposed to be dominated by interlayers. However, the results still suggest that ternary system has better potential in achiveing long-term stable OSCs.

Corresponding device physics analyses are done altogether while device efficiency measurement. The exciton dissociation (*η*_diss_) and charge collection efficiencies (*η*_coll_) of the devices were investigated through *J*_ph_ vs *V*_eff_ curves plotted in Fig. S14a and the corresponding efficiencies presented in Table S2, the addition of BTP-BO-3FO can enhance the saturated current density (*J*_sat_) in both CF- and *o*-XY-processed devices and slightly improvement in *η*_diss_ was observed in *o*-XY-processed devices which is consistent with changes in *J*_SC_ and FF values. Also, the charge recombination mechanisms of the devices are investigated by figuring out the relationship between illumination intensity and *V*_OC_/*J*_SC_ plotted in Fig. S14b, c. The n values calculated from *V*_OC_ vs light intensity lines are 0.934, 0.994, 1.13, 0.971 and 0.974, respectively. These results indicate that introducing proper amount of BTP-BO-3FO can reduce the surface recombination dominance, but overdosing it could result in more severe trap-assisted recombination [39–41]. The slopes of *V*_OC_ vs light intensity curves are calculated to be 0.987, 0.983, 0.977, 0.978 and 0.976. They are all close to 1, indicating that the bimolecular recombination is generally reduced. The slight change of their values could be the result of altered charge mobility, instead of unfavorable recombination dynamics, according to subsequent charge transport and recombination analyses [42–44]. The energy transfer existence is confirmed by using photoluminescence (PL) measurements (Fig. S14d) [45]. Accordingly, clear enhancement of PL signal intensity of blend acceptor films compared to pure films can be attributed to energy transfer between BTP-eC9 and BTP-BO-3FO. Meanwhile, the series and shunt resistances (*R*_series_, *R*_shunt_) are also investigated via resistance vs applied voltage (*R*_diff_-*V*) curves (Fig. S15). Principally high *R*_shunt_ and low* R*_series_ are desired, and accordingly obtained data are consistent with the *FF* variation tendency. Furthermore, we also present the corrected voltage (*V*_cor_) based *J–V* characteristics in Fig. S15 for reference [46].

### Morphology Analysis

The device performance is supposed to be tightly correlated to the film morphology, especially for our cases that two different solvents brought to different ternary design’s efficiency improvement effect. Before analyzing blend films, the acceptor-only films are focused on, since structurally similar acceptors are supposed to have good miscibility, thus interactive crystallization tuning effect. Figuring out what happens within acceptors is helpful to understanding overall morphology change of active layers. Herein, both UV–Vis absorption profiles and PL spectra of those films are presented in normalized way. The fitting methods are all proposed by previous studies [47–49]. The green-shaded region represents the spectroscopical contribution from S1 ordered aggregation phase in film, and the organged part comes from S1 amorphous phase. The S2 and S3 contributions are marked gray and blue, respectively. Starting with CF-processed acceptor films related UV–Vis spectra (Fig. S16a), the green-shaded part is composed by two peaks, referring to S0 → S1 electronic transition, while the orange-shaded region is considered to represent the amorphous state. According to the results of UV–Vis spectrum fittings, blending BTP-BO-3FO with BTP-eC9, especially when its content becomes high, more amorphous states are induced, under CF processing. BTP-BO-3FO is intrinsically with strong amorphous property, though it cannot be found in UV–Vis analysis, but clearly shown in PL fitting (since it is more emissive than BTP-eC9). As for PL signals for 1:0.3 and 0.7:0.6 weight ratio films in Fig. S16b, the reduced amorphous state might not be contradictory to their UV–Vis spectra, since there exists energy transfer between two materials, which is supposed to bring more emission from BTP-eC9’s ordered phase.

Subsequently more attention is paid on two *o*-XY-processed films, whose UV–Vis and PL analyses are all presented in Fig. S16c. Compared to its CF-processed counterpart, *o*-XY cast neat BTP-eC9 film is more ordered in 0–0 vibrational part, but less ordered in 0–1 region, which would possibly make its interaction with BTP-BO-3FO different here. Interestingly, 0.3/1.3 ratio’s BTP-BO-3FO does increase the ordered peaks while amorphous state’s contribution becomes significantly weaker. This is also observed in PL spectra, where the emission from amorphous state is nearly erased out.

**Fig. 3:**
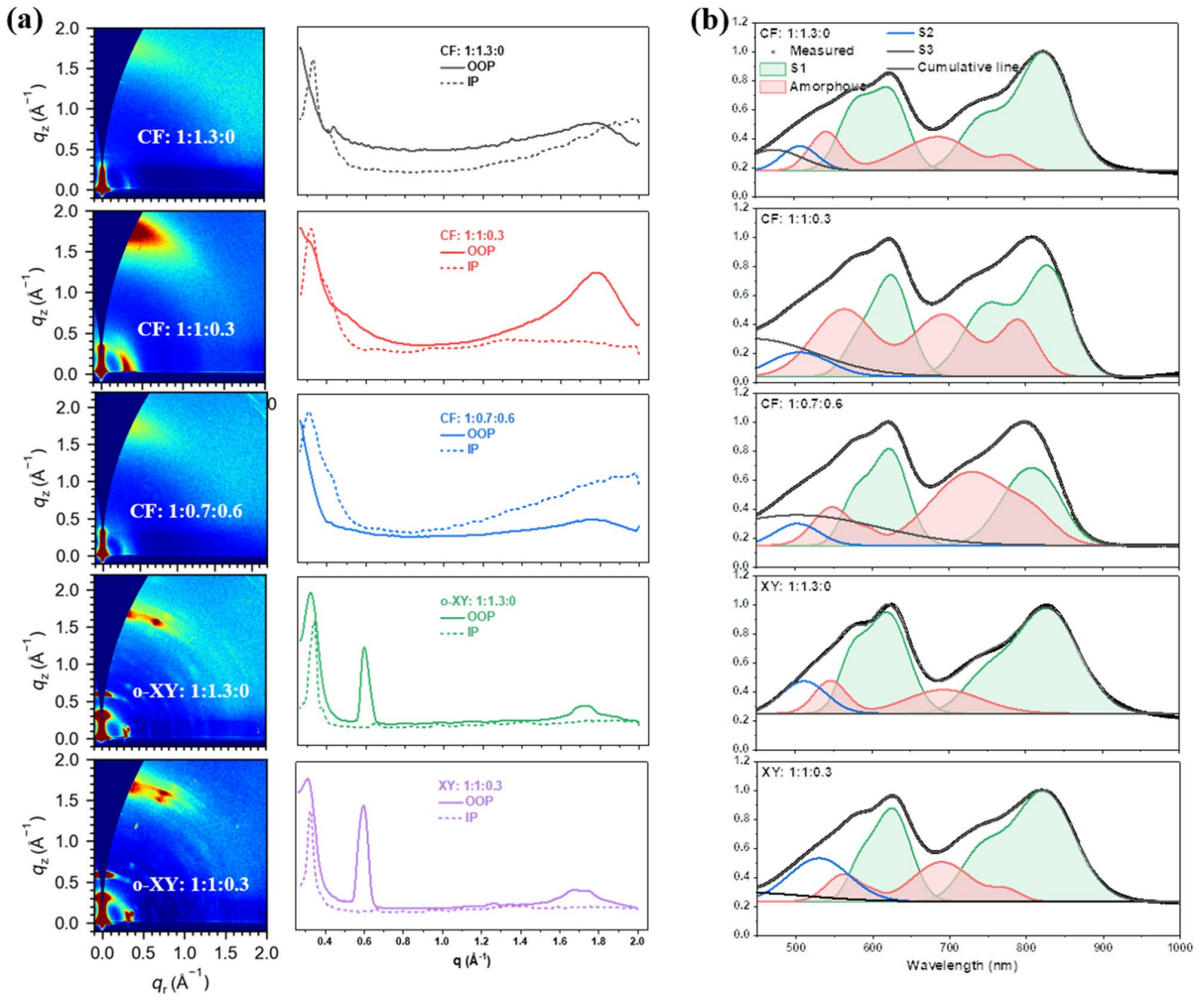
Blend film morphology analysis: **a** 2D GIWAXS patterns and relative line-cuts. **b** UV–Vis spectra with gaussian fitting results

Then we turn on the focus to D/A blend films to further reveal the different morphology evolution upon ternary blend films caused by solvent variation. Beginning at the nanometer scale molecular packing, the grazing incidence wide-angle X-ray scattering (GIWAXS) tests are utilized [50–53]. The 2D patterns and extracted line-cuts are displayed in Fig. 3a, and the fitted parameters for π–π stacking are demonstrated in Table S3. Within CF-cast films, the coherence length (CL) values for (010) peaks are very significantly reduced along with the increase of BTP-BO-3FO’s content. Fewer orderly packed molecules through π–π interaction is negative to the charge transport and then decreased *FF*. In parallel, the *o*-XY films display similar variation trend, yet weaker degree of crystalline ordering decrease. After the comparison of crystallite order, the proportion of ordered phase and amorphous phase is further studied, also enabled by UV–Vis spectrum fitting. The green shaded regions separately represents UV–Vis absorption from ordered donor and acceptor(s), while the organge patterns here are the sum of amorphous phases of both donor and acceptor(s). According to the results shown in Fig. 3b, adding BTP-BO-3FO significantly damages the ratio of ordered phase (lower relative peak height and disappeared 0–1 peaks for donor and acceptors), while improves amorphous state’s proportion, which is supposed to reduce the chare transport ability, as well. In contrast, ternary film cast from *o*-XY does not lead to clearly order/amorphous phase’s content change. With a better maintained film morphology, and tuned energy level distribution, the *o*-XY-processed ternary devices rationally exhibit as good *FF* as that of binary counterparts.

To confirm the charge transport deduction, the hole and electron mobility is evaluated by fabricating a series of hole-only and electron-only devices, that is, the space charge limited current (SCLC) method. As shown in Fig. S17 and Table S4, obviously improvement in both hole and electron mobility (also their balance) in *o*-XY-processed ternary systems, totally different from CF-processed devices, which echoes the above morphology analysis.

**Fig. 4:**
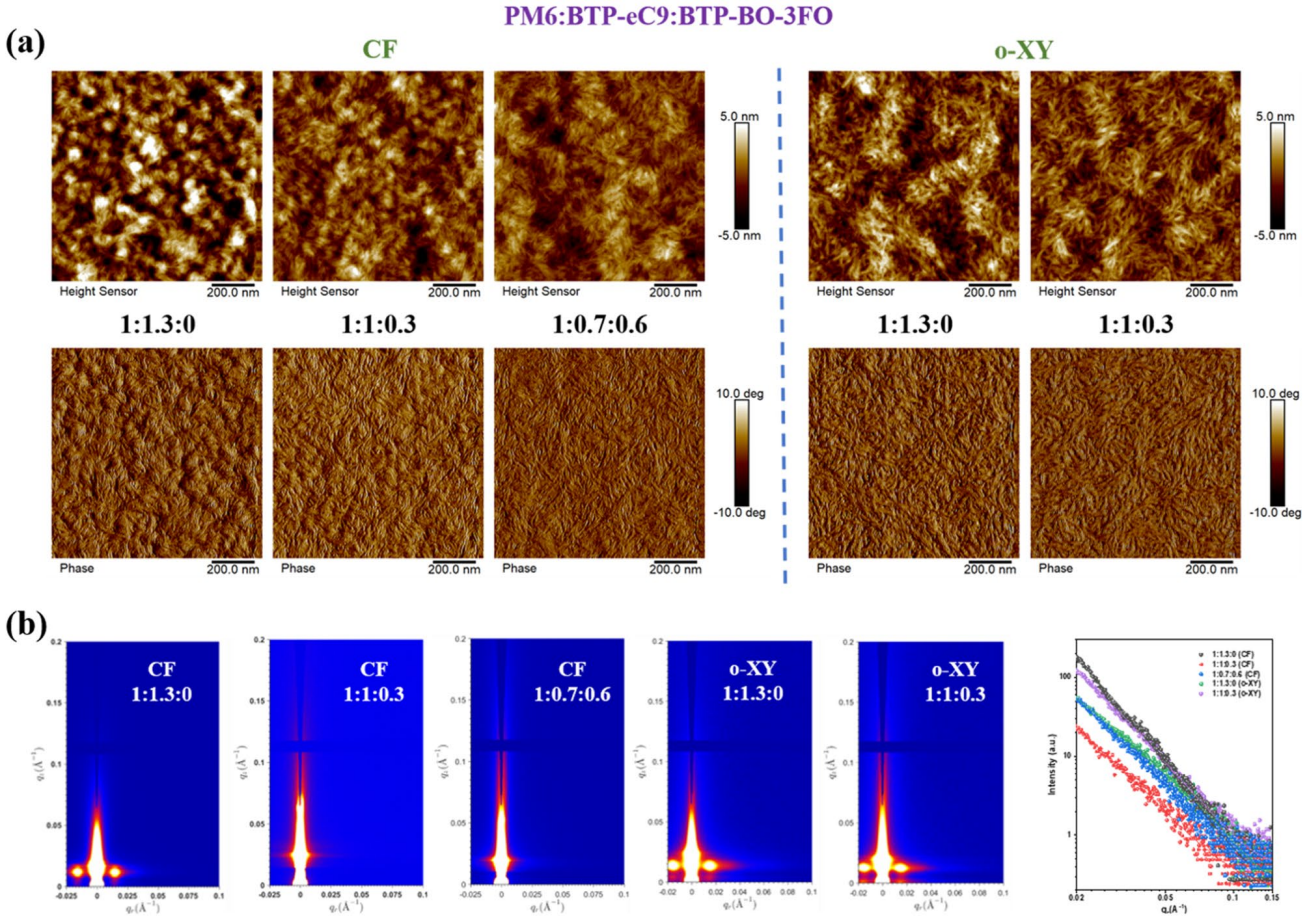
**a** Blend AFM and **b** GISAXS

Next, larger nanometer scale (10 nm to 50 nm) film morphology investigation is supported by atomic force spectroscopy (AFM) and grazing incidence small-angle X-ray scattering (GISAXS) experiments [54–56]. The obtained height/phase images, 2D intensity patterns, line-cuts and fitting results are all provided in Fig. 4. The AFM height and phase images both suggest *o*-XY-processed PM6:BTP-eC9 has more favorable interpenetrating fibrils compared to aggregates contained morphology of the CF cast counterparts, consistent with our previous finding. Then it is notable that ternary films cast from CF exhibit very clearly reduced phase separation marks, also considered as a phenomenon of decreased ordered phase. Meanwhile, the *o*-XY-processed 1:1:0.3 film presents very similar to its binary control, referring to a better-kept morphology.

The quantitative study is then carried out based on GISAXS calculation results that are summarized in Table 2. Obviously, *o*-XY treatment results in more interpenetrating morphology than CF does, as both amorphous state length scale (ξ) and cluster radius of aggregates (*R*_g_) values are lower there. As the rise of *R*_g_ values with the addition of BTP-BO-3FO in CF-processed active layers, and the figured out reduced ordered phase, its amount of PM6 or acceptor aggregates shall be reduced, which is consistent to AFM images. However, the *R*_g_ values treated by *o*-XY are getting smaller in ternary blend film, and thus more small-size aggregates with decent purity show up, which is beneficial to charge generation and transport. This is also consistent with the coherence length (*η*) results, and fractal dimension of acceptor (D) values: o-XY-processed films locate at lower level [57].

**Table 2:** GISAXS fitting results

PM6:BTP-eC9:BTP-BO-3FO	*ξ* (nm)	*η* (nm)	*D*	*R*_g_ (nm)
*CF processed*				
1:1.3:0	21.6	11.3	3.0	23.9
1:1:0.3	15.2	9.9	3.4	26.6
1:0.7:0.6	15.8	12.1	3.2	31.2
*o-XY processed*				
1:1.3:0	7.1	7.9	2.3	15.4
1:1:0.3	7.5	5.6	2.2	10.4

To obtain more understanding from thermodynamic view, the powerful tool, UV–Vis absorption measurement is again utilized [58–60]. The absorption deviation metrics (DM_T_) can be obtained from different temperature annealing enabled normalized absorptions of all films (Fig. S18), and the extracted information presented in Fig. S19 locates the glass transition points (*T*_g_). The CF cast neat BTP-eC9 film demonstrates an 80 °C *T*_g_, which is lowest among all films investigated here, and in consistence with the previous report. However, its *o*-XY-processed counterpart has a much higher* T*_g_ of 107 °C. Considering the prepared concentration of BTP-eC9 in *o*-XY is twice higher than that in CF, while the film drying time is also much longer, this big *T*_g_ value gap is due to more condensed film enabled by *o*-XY. Therefore, BTP-BO-3FO (*T*_g_ = 110 °C processed by CF) easily change the host system’s *T*_g_ if it is cast from CF, but the *T*_g_ values of all o-XY-processed films are stabilized in the range of 106–110 °C. In addition, the slopes of two stages for these films demonstrate that o-XY-processed films contain stabler molecular aggregation states, probably due to its longer film drying duration leads to a less non-equilibrium morphology state. This could also explain why the *T*_g_ varied limitedly for binary and ternary films cast from o-XY.

### Exciton Behavior Evaluation

**Fig. 5:**
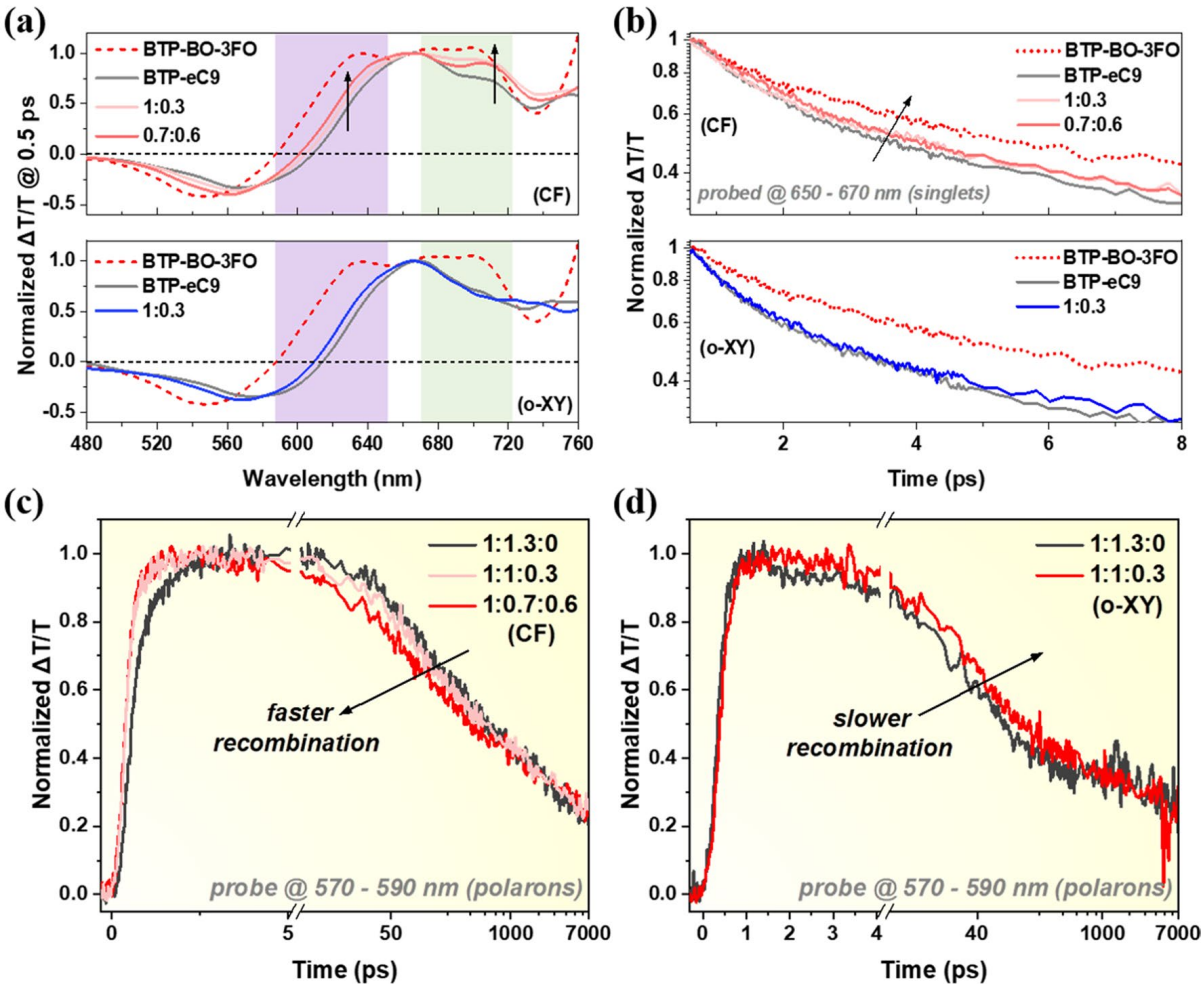
**a** TAS spectra @ 0.5 ps after 800 nm pump laser for acceptor-only films. **b** Singlet excitons GSB signal decay kinetics of acceptor-only films. Exciton generation and recombination kinetics of **c** CF- and **d** *o*-XY-processed blend films

Finally, the charge behavior is investigated by the femtosecond transient absorption spectroscopy (fs-TAS) technology, as an important supplementary study aside from the morphology analysis.^62,63^ The TAS representative spectra of acceptor-only and blend films are all shown in Figs. S20 and S21, inserted with their 2D color maps. Figure 5a shows the TAS spectra at 0.5 ps after pumping to represent the photogenerated singlet excitons. It can be observed that the features of BTP-eC9 and BTP-BO-3FO are of great difference which are also inferable from their relative absorption spectra. Under CF processing, their alloy films spectral shape changes relative to the BTP-BO-3FO content, as highlighted in both purple and green shaded regions. In contrast, o-XY processing with 0.3/1.3 ratio BTP-BO-3FO barely changes with BTP-eC9 host film indicative that the BTP-eC9’s superior property is well maintained. It is also supported by the singlet exciton lifetime variation plotted in Fig. 5b, where the decay rates of CF-processed alloy films are at the middle part of two neat films, but that of o-XY cast bi-acceptor film is almost identical to the pure BTP-eC9 counterpart. This can be attributed to BTP-BO-3FO chromophore is closer to BTP-eC9 chromophore in o-XY-processed films with suitable PL and absorption overlaps, thereby suggestive to enable energy transfer. The polarons of blend films are detected at 570–590 nm and analyzed to represent the sub-ns scale recombination kinetics and the ultrafast charge transfer process. The lines are then plotted in Fig. 5c, d for CF- and o-XY-coated active layers, respectively. The polarons generation rate appears faster with BTP-BO-3FO content upon CF processing while it remains dominated by BTP-eC9 for o-XY processing, complimenting that BTP-eC9 superior characteristics is well maintained when o-XY is the precursor solvent. Meanwhile, we can see promoting BTP-BO-3FO’s content monotonically increases the recombination rate, which is consistent with *FF* loss in CF-processed devices. On the other hand, the addition of BTP-BO-3FO does not increase the recombination rate up upon o-XY processing. Instead, it is slightly slower which is well consistent with the observed *FF* improvements. Note the recombination decay curves might not be very significant from each other in these two graphs; however, such situations take place from time to time in recent reports [63–67]. More importantly, the yielded tendency is a good explanation for device performance variation.

## Conclusions

In summary, by developing a novel fluorine- and methoxy-co-substituted terminal group and applying asymmetric substitution strategy, a SMA called BTP-BO-3FO is synthesized as an effective guest material in pursuing the PCE of host system PM6:BTP-eC9 processed by both CF and o-XY. Furthermore, the use of BTP-BO-3FO brings different parameter variation features in two solvent-processed devices. Morphological analyses (GIWAXS, GISAXS, AFM, UV/PL fitting) and photo-physics characterizations reveal the different packing/aggregation ordering changings shall be the explanation. This work not only provides a successful material design case that helps ternary blend achieving higher PCEs, (especially 19.24% for green-solvent processed one), but also a reminder that solvent selection has strong impact on ternary strategy’s effect, as well as a research template of how to analyze these differences.

## Supplementary Information

Below is the link to the electronic supplementary material.

Supplementary file1 (PDF 1837 kb)
