# Supplementary material for: Step-by-Step Modulation of Crystalline Features and Exciton Kinetics for 19.2% Efficiency Ortho-Xylene Processed Organic Solar Cells
Source: Nanomicro Lett. 2023 Nov 23;16:30. doi: 10.1007/s40820-023-01241-z (PMC10667184; doi:10.1007/s40820-023-01241-z)
Supplement: Supplementary file 1 — Supplementary file1 (PDF 1837 kb) [file 40820_2023_1241_MOESM1_ESM.pdf]

Supporting Information for

Step-By-Step Modulation of Crystalline Features and Exciton Kinetics for 19.2% Efficiency Ortho-Xylene Processed Organic Solar Cells

Bosen Zou[1, #], Weiwei Wu[1, #], Top Archie Dela Peña[1, 3, 4, #], Ruijie Ma[2, *], Yongmin Luo[3], Yulong Hai[3], Xiyun Xie[2], Mingjie Li[4], Zhenghui Luo[5,], Jiaying Wu[3, *], Chuluo Yang[5], Gang Li[1], He Yan[1, *]

[1]Department of Chemistry Department of Chemistry and Hong Kong Branch of Chinese National Engineering Research Center for Tissue Restoration and Reconstruction, The Hong Kong University of Science and Technology, Clear Water Bay, Hong Kong, P. R. China

[2]Department of Electronic and Information Engineering, Research Institute for Smart Energy (RISE), Guangdong-Hong Kong-Macao (GHM) Joint Laboratory for Photonic-Thermal-Electrical Energy Materials and Devices, The Hong Kong Polytechnic University, Hung Hom, Kowloon, Hong Kong, 999077, P. R. China

[3]The Hong Kong University of Science and Technology, Function Hub, Advanced Materials Thrust, Nansha 511400, Guangzhou, P. R. China

[4]Department of Applied Physics, The Hong Kong Polytechnic University, Hong Kong, P. R. China

[5]Shenzhen Key Laboratory of New Information Display and Storage Materials, College of Materials Science and Engineering, Shenzhen University, Shenzhen, 518060 P. R. China

[#] Bosen Zou, Weiwei Wu, and Top Archie Dela Peña share the co-first authorship in this work.

*Corresponding authors. E-mail: ruijie.ma@polyu.edu.hk (Ruijie Ma), hyan@ust.hk (He Yan), jiayingwu@ust.hk (Jiaying Wu), zhhuiluo@szu.edu.cn (Zhenghui Luo)

S1 Synthesis

All chemicals, unless otherwise specified, were purchased from Aldrich or other commercial resources and used as received. The starting material (3-Fluoro-4-methoxybenzoicAcid and BTP-BO-CHO) is commercially available. Toluene and THF were distilled from sodium benzophenone under nitrogen before using. ^{1}H NMR spectra were recorded on a Bruker AV-400 MHz NMR spectrometer. Chemical shifts are reported in parts per million (ppm, δ). ^{1}H NMR spectra were referenced to

tetramethylsilane (0 ppm) for $CDCl_3$. Mass spectra were collected on a MALDI Micro MX mass spectrometer, or an API QSTAR XL System. The synthesis steps of the end group of IC-FOMe and the SMAs are similar as our previous report, the corresponding synthesis information are shown below.

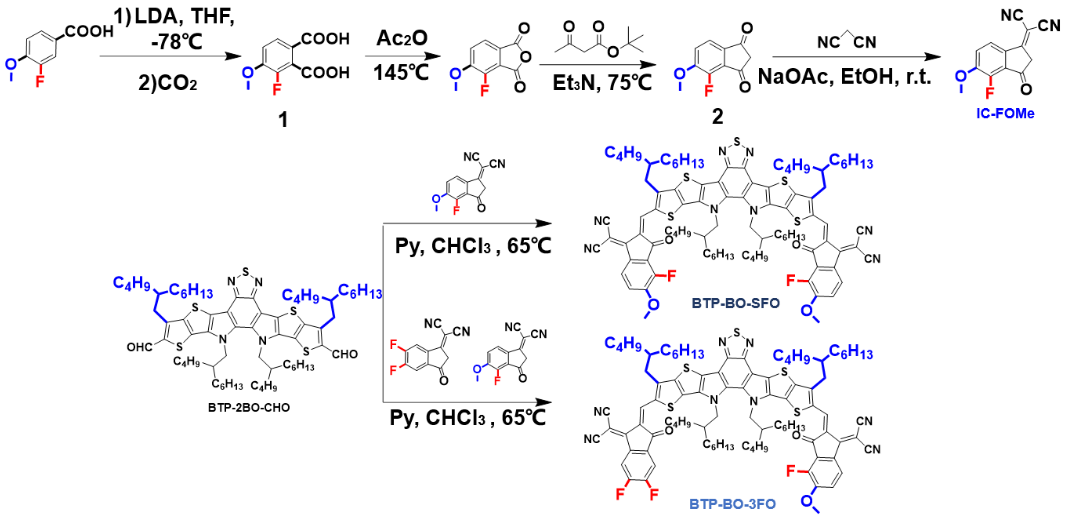

Scheme S1 Synthesis of IC-FOMe, BTP-2BO-SFO, BTP-2BP-3FO

Synthesis of **Compound 1**

Compound 1 (10 g, 58.8 mmol) was dissolved in distilled THF (50 mL), and then 2.0 M lithium diisopropylamide in hexane (59 mL, 118 mmol) was added dropwise under N_2. The reaction was stirred at -78℃ for 3 h and then CO_2 gas was added, The reaction mixture was returned to room temperature and stirred overnight. The mixture was poured into water, and acidified to pH 1–2 by addition of the diluted HCl and extracted with EA for three times. The combined organic phase was washed with water followed by brine. Then the solution was dried over Na_2SO_4 and concentrated under reduced pressure. The residue as light yellow solid was used directly without further purification.

Synthesis of **Compound 2**

Compound 2 (2.5 g, 4.31 mmol) was dissolved in acetic anhydride (15 mL), the reaction was stirred at 145 °C refluxed for 2.5 h. Then reaction mixture was cooled to room temperature, triethylamine (8 mL) and tert-butyl acetoacetate (1.22 g, 7.75 mmol) were added dropwise and the reaction was stirred at 75 °C overnight. The reaction mixture was poured over ice with HCl and extracted with DCM, The combined organic phase was washed with water followed by brine. Then the solution was dried over Na_2SO_4 and concentrated under reduced pressure. The **Compound 2** as brown solid was used directly without further purification.

Synthesis of **IC-FOMe**

Compound 2 (1 g, 5.15 mmol), malononitrile (0.690 g, 10.4 mmol) were dissolved in

25 mL absolute ethanol, and then anhydrous sodium acetate (852.8 g, 10.4 mmol) was added while stirring under room temperature. After 12 h, the mixture was poured into water, and acidified to pH 1–2 by addition of the hydrochloric acid. Then reaction mixture was extracted by DCM three times and dried over Na_2SO_4. The crude product was purified by silicon chromatography with DCM to get pure product IC-FOMe (0.73 g, 59%) . ^{1}**H NMR** (400 MHz, $CDCl_3$) δ 7.76 (d, J = 8.3 Hz, 1H), 7.42 (t, J =7.6 Hz, 1H), 4.06 (s, 3H) , 3.74 (s, 2H). MS (CI) [M] calcd. for ($C_{13}H_7FN_2O_2$): 242.21. Found: 243.06.

Synthesis of **BTP-2BO-SFO**

BTP-2BO-CHO (300 mg, 0.257 mmol), **IC-FO** (155.54 mg, 0.642 mmol) were dissolved in absolute chloroform (20 mL), and pyridine (2 mL) were added. The mixture was deoxygenated with nitrogen for 30 min and then refluxed for 6 h. After cooling to room temperature, the mixture was poured into methanol (150 mL) and filtered. The residue was purified by column chromatography on silica gel using petroleum ether/DCM (1:2) as eluent, yielding a dark blue solid and recrystallization through MeOH/DCM for two times to obtain **BTP-2BO-SFO** (270 mg, 65%). ^{1}**H NMR** (400 MHz, $CDCl_3$) δ 9.14 (s, 2H), 8.53 (d, J = 8.7 Hz, 2H), 7.31 – 7.27 (m, 2H), 4.76 – 4. (m, 4H), 4.06 (s, 6H), 3.20 (d, J = 7.3 Hz, 4H), 2.21 – 2.08 (m, 4H), 1.49 – 0.53 (m, 88H). MS (CI) [M] calcd. for ($C_{94}H_{112}F_2N_8O_4S_5$): 1616.28. Found: 1616.76.

Synthesis of **BTP-2BO-3FO**

BTP-2BO-CHO (300 mg, 0.257 mmol), **IC-FO** (63 mg, 0.257 mmol), **IC-2F** (59 mg, 0.257 mmol) were dissolved in absolute chloroform (20 mL), and pyridine (2 mL) were added. The mixture was deoxygenated with nitrogen for 30 min and then refluxed for 6 h. After cooling to room temperature, the mixture was poured into methanol (200 mL) and filtered. The residue was purified by column chromatography on silica gel using petroleum ether/dichloromethane (1:2) as eluent, yielding a dark blue solid and recrystallization through MeOH/DCM for two times to obtain **BTP-2BO-3FO** (145 mg, 35%). ^{1}**H NMR** (400 MHz, $CDCl_3$) δ 9.14 (d, J = 9.9 Hz, 2H), 8.78 (s, 1H), 8.52 (d, J = 8.6 Hz, 1H) , 7.95 (s, 1H), 7.30 – 7.24 (m, 1H), 4.78 (d, J = 7.5 Hz, 4H), 4.06 (s, 3H), 3.17 (d, J = 4.6 Hz, 4H), 2.07 (dd, J = 26.6, 15.0 Hz, 4H), 1.51 – 0.58 (m, 88H). MS (CI) [M] calcd. for ($C_{93}H_{109}F_3N_8O_3S_5$): 1604.24. Found: 1603.73.

S2 Characterization

UV-vis absorption spectra were measured using a Shimadzu UV-2500 recording spectrophotometer. AFM measurements were obtained by using a Dimension Icon AFM (Bruker) in a tapping mode. The grazing incidence small/wide angle X-ray scattering (GISAXS/GIWAXS) measurements were carried out with a Ganesha SAXSLAB laboratory instrument using a CuKα X-ray source (8.05 keV, 1.54 Å) and a Pilatus 300K detector. The samples for GIWAXS/GISAXS measurements were fabricated on silicon substrates using the same recipe as for the devices. The incident

angle was 0.4° for GISAXS and 0.2° for GIWAXS measurements, respectively. The sample to detector distance (SDD) was set to 1045 and 95 mm for GISAXS and GIWAXS measurement. For the GISAXS images, the DPDAK software was applied to extract the polymer scattering signals. The transformation to q-space, radial cuts for the in-plane and out-of-plane analysis and azimuthal cuts for the orientation analysis were processed by the MATLAB-based package GIXSGUI.

S3 SCLC Measurements

The electron and hole mobility were measured by using the method of space-charge limited current (SCLC) for electron-only devices with the structure of ITO/ZnO/active layer/PFN-Br-MA/Ag and hole-only devices with the structure of ITO/PEDOT:PSS-TA/active layers/MoO_x/Ag. The charge carrier mobility was determined by fitting the dark current to the model of a single carrier SCLC according to the equation: $J = 9\varepsilon_0\varepsilon_r\mu V^2/8d^3$, where J is the current density, d is the film thickness of the active layer, μ is the charge carrier mobility, ε_r is the relative dielectric constant of the transport medium, and ε_0 is the permittivity of free space. $V = V_{app} - V_{bi}$, where V_{app} is the applied voltage, V_{bi} is the offset voltage. The charge carrier mobility was calculated from the slope of the $J^{1/2} \sim V$ curves. The thickness of target layer is well controlled identical to that of PV's active layer.

S4 Analysis of J_{ph} vs V_{eff} Relationships

The definition of J_{ph} is the current density under illumination (J_L) minus the dark current density (J_D), and V_0 refers to the voltage value when J_{ph} = 0. Accordingly, $V_{eff} = V_0 - V_{appl}$, where V_{appl} represents applied voltage, has a clear meaning. Importantly, when V_{eff} reaches a high value (> 2V) it is normally believed that generated excitons are fully collected, in which J_{ph} is equal to saturated current density (J_{sat}). Then, we can calculate J_{SC}/J_{sat} and J_{max}/J_{sat} to describe exciton dissociation (η_{diss}) and charge collection (η_{coll}) efficiency. J_{max} is the J_{ph} at the maximal output point.

S5 UV-vis and PL Spectra Fitting Method

UV–vis and PL spectra are modelled as linear superpositions of basis spectra from individual absorbers:

A =Σi bi×i (S1)

where A = f(E) is the decadic absorbance, bi = f(E) is the (unitless) basis spectrum of material i, which depends on the irradiated energy E, and si is the spectral weight (in units of eV). The index i ∈ {D, A} comprises the donor and acceptor materials, respectively, if applicable. The basis spectra for each material are given as linear superpositions of sub-bands whose shapes are given by hyperparameters that contain morphology information:

bi =Σj bi,j (ai,j, wi,j, ci,j, dc,i,j, hi,j, ni,j), (S2)

where the index j ∈ {1o, 1a, 2, 3} comprises contributions from the three lowest energetic-allowed optical transitions. For j = 1, we distinguish between contributions

from an ordered phase and an amorphous phase (suffixes ‘o’ and ‘a’, respectively). This picture has been shown to yield good results in P3HT (refs. 55, 56), PM6 (ref. 32) and Y6 (ref. 33). We model electron–phonon coupling by assuming one effective vibronic progression as a superposition of Gaussian bands of same width wi,j and fixed energy offset dci,j against the energy ci,j of the (0–0) vibronic transition57 for a given electronic transition and the individual spectral weight given by the Huang–Rhys factor, hi,j, of this effective progression. For donor polymers, we adopt the model of weak H aggregates (‘Spano model’)58 in which the (0–0) vibronic transition is suppressed by a factor ni,j with respect to the other vibronic transitions of the given progression. We use nonlinear regression (function curve_fit of the Python library scipy) to fit the experimental absorption spectra by tuning the hyperparameters in equation (2) and Penrose pseudo matrix inversion (using scipy function lsq_linear) to obtain the overall spectral weights in equation (1). However, because there is linear dependence between

si and ai,j, we need to fix at least one of these parameters. Thus, we follow the convention that the ordered region of the lowest energetic electronic transition of each material has unity spectral weight:

ai,1o ≡ 1 (3)

Furthermore, due to spectral congestion in the absorption spectra, we reduced the number of free hyperparameters by fixing nD,1a = nD,1o = 0.5, which is a typical value for donor polymers, and by fixing nA,1a = nA,1o = 1 because the acceptor systems of this work are dominated by strong J aggregates rather than weak H aggregates as would be required by the Spano model.

S6 Transient Absorption Spectroscopy

Transient absorption spectroscopy (TAS) was measured with an amplified Ti:sapphire femtosecond laser (800 nm wavelength, 50 fs, 1 kHz repetition; Coherent Libra) and a Helios pump/probe setup (Ultrafast Systems). The 400 nm pump pulses with a pump fluence of 0.5 or $< 3\ \mu J/cm^2$ were obtained by frequency doubling the 800 nm fundamental regenerative amplifier output. The white-light continuum probe pulses were generated by focusing a small portion of the regenerative amplifier’s fundamental 800 nm laser pulses into a 2 mm sapphire crystal.

Supplementary Figures and Tables

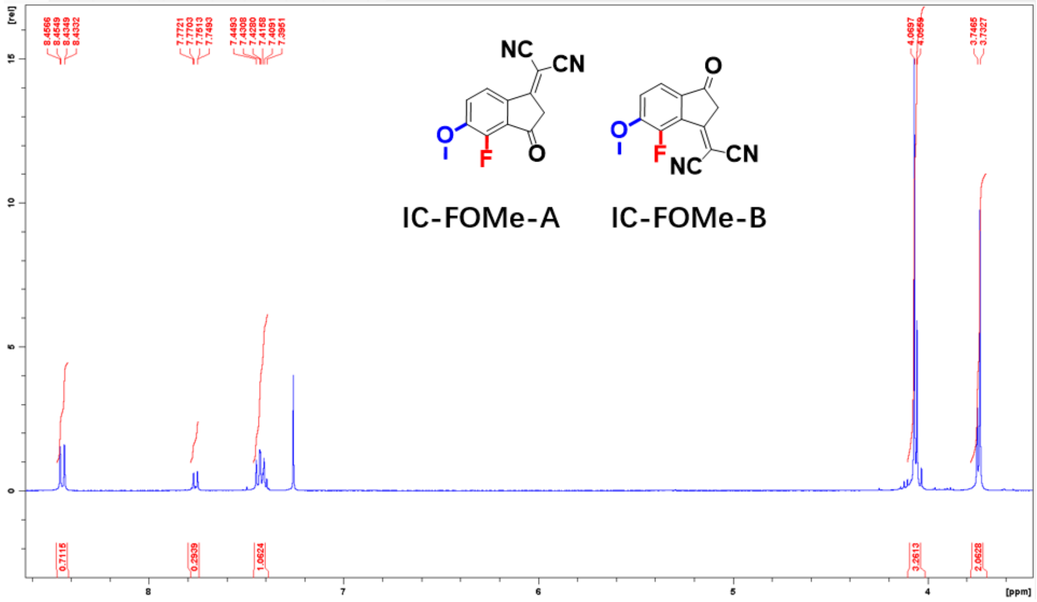

Fig. S1 ^{1}H NMR spectrum of blend **IC-FOMe** (400 MHz, $CDCl_3$)

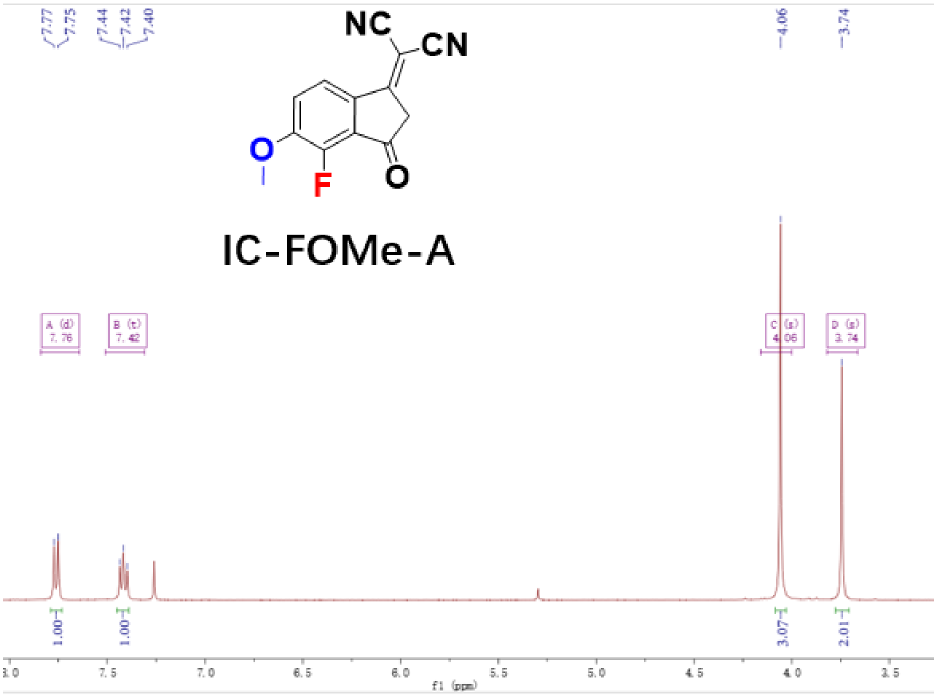

Fig. S2 ^{1}H NMR spectrum of pure **IC-FOMe-A** (400 MHz, CDCl3)

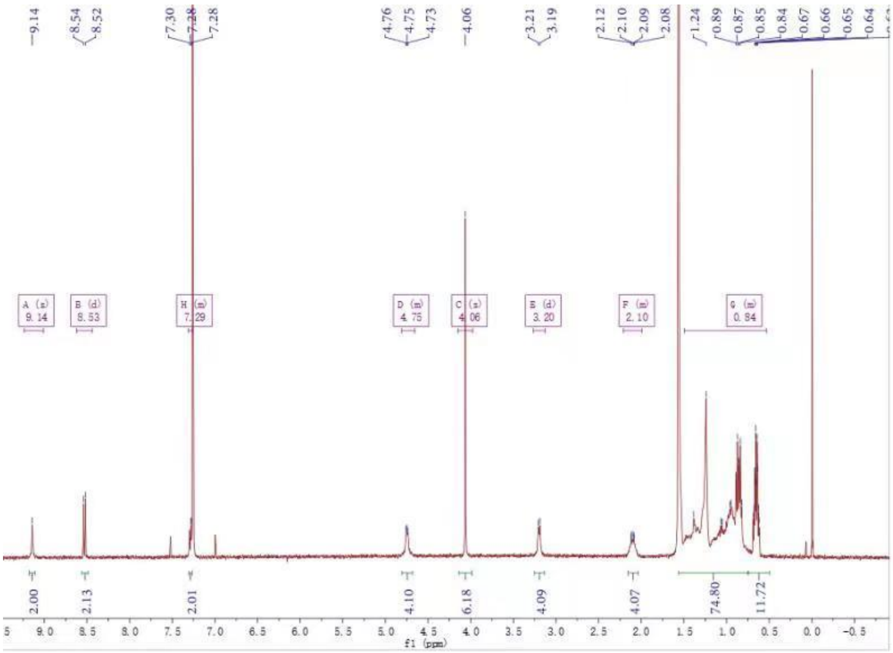

Fig. S3 ^{1}H NMR spectrum of **BTP-BO-SFO** (400 MHz, CDCl3)

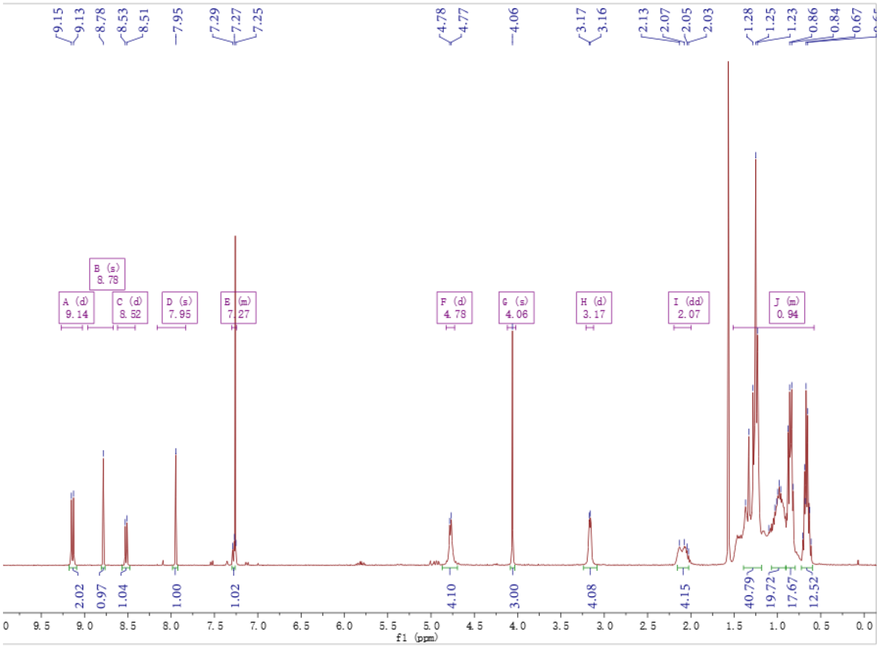

Fig. S4 ^{1}H NMR spectrum of **BTP-BO-3FO** (400 MHz, CDCl3)

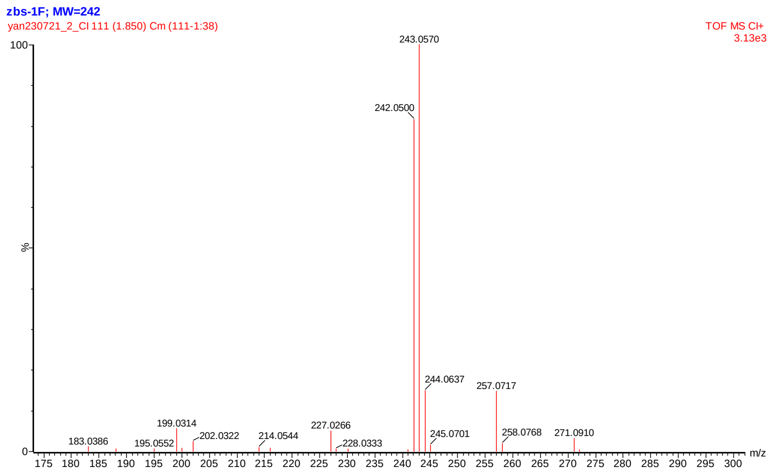

Fig. S5 MS spectrum (MALDI-TOF) of compound **IC-FOMe**

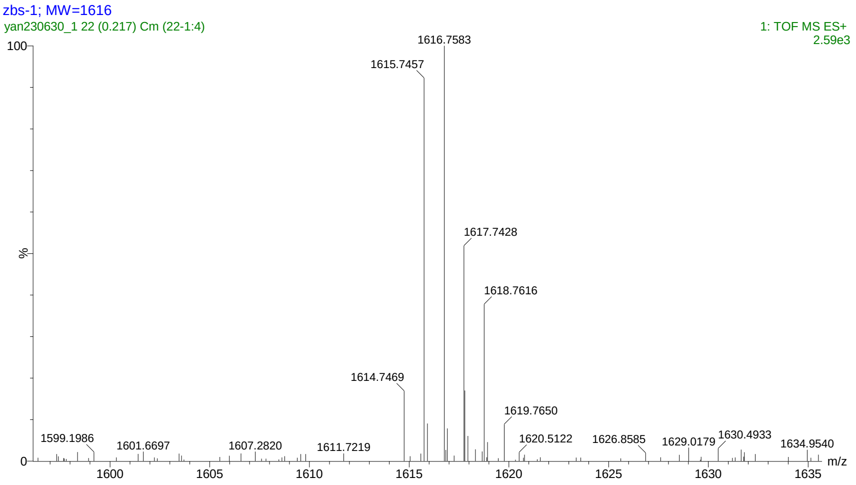

Fig. S6 MS spectrum (MALDI-TOF) of **BTP-BO-SFO**

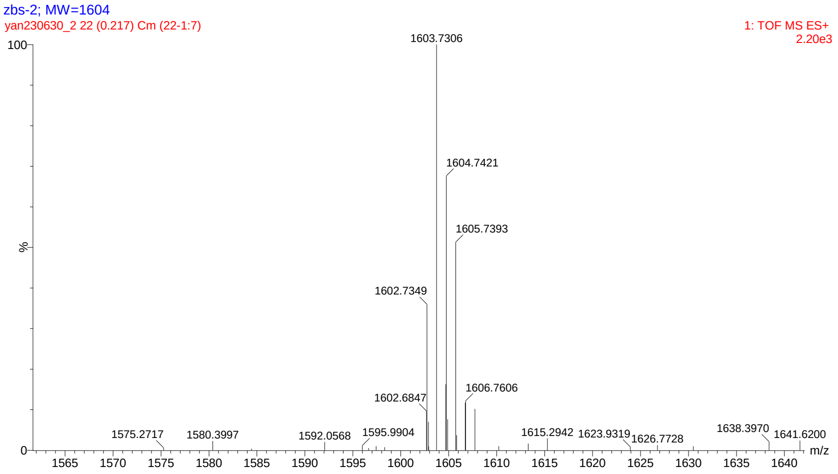

Fig. S7 MS spectrum (MALDI-TOF) of **BTP-BO-3FO**

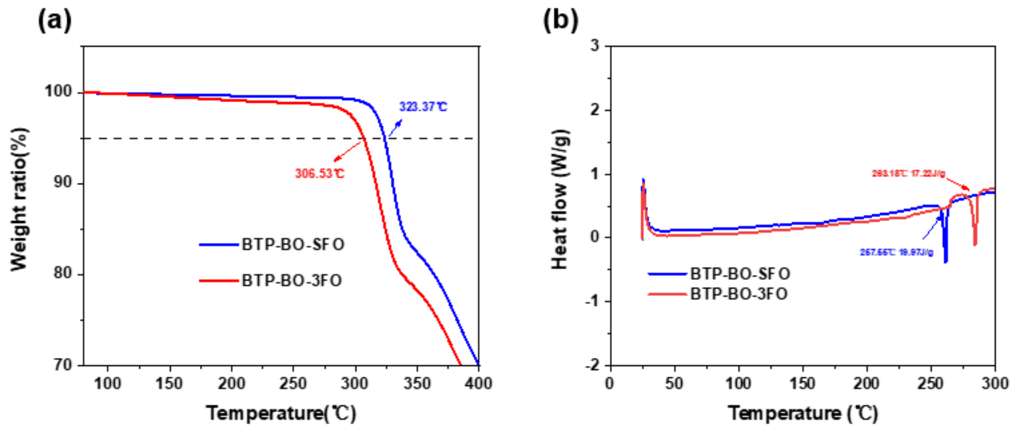

Fig. S8 (**a**) Thermogravimetric analysis (TGA) curves of BTP-BO-SFO and BTP-BO-3FO. (**b**) The differential scanning calorimetry (DSC) curves of BTP-BO-SFO and BTP-BO-3FO

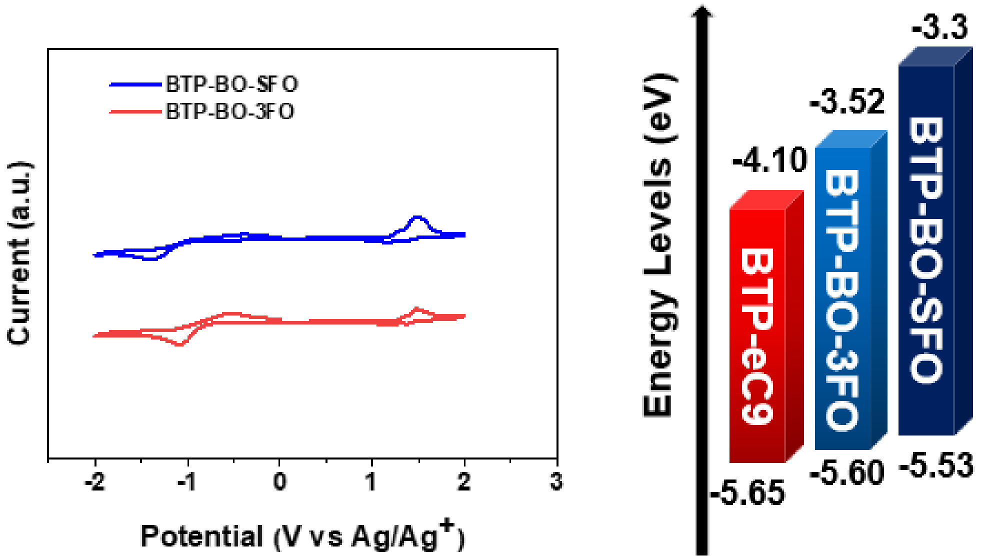

Fig. S9 CV, energy level distribution

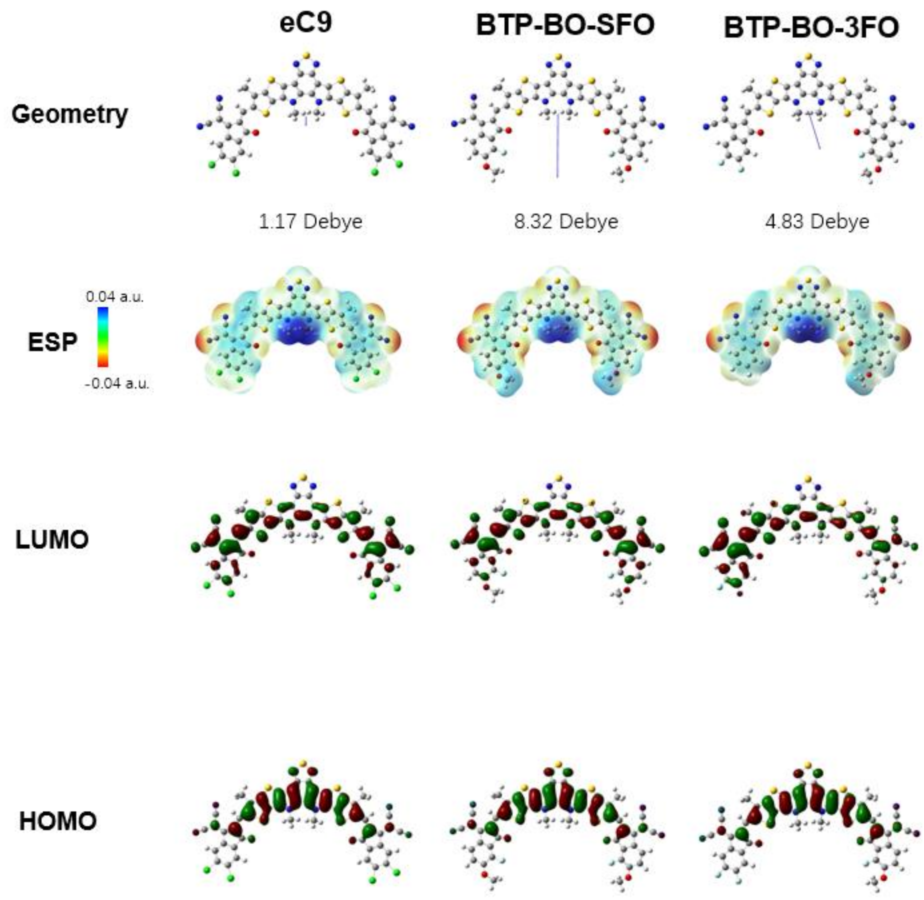

Fig. S10 DFT data of **eC9, BTP-BO-SFO and BTP-BO-3FO**

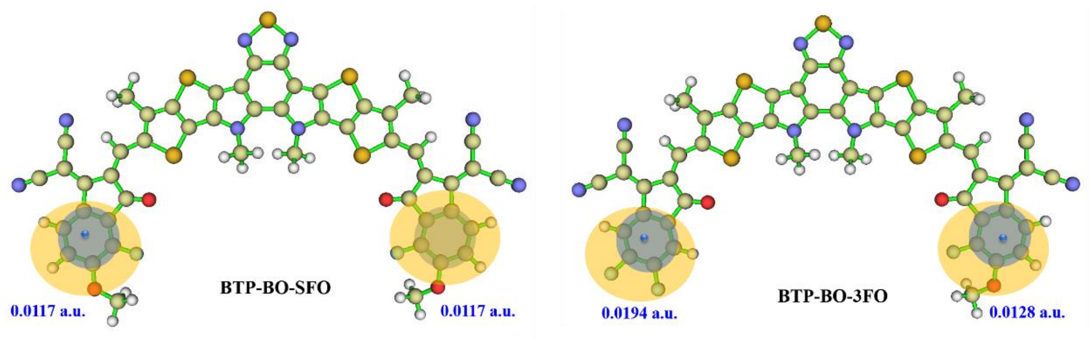

Fig. S11 The calculated optimal conformation and ESP average value of benzene ring in the end groups for BTP-BO-SFO and BTP-BO-3FO

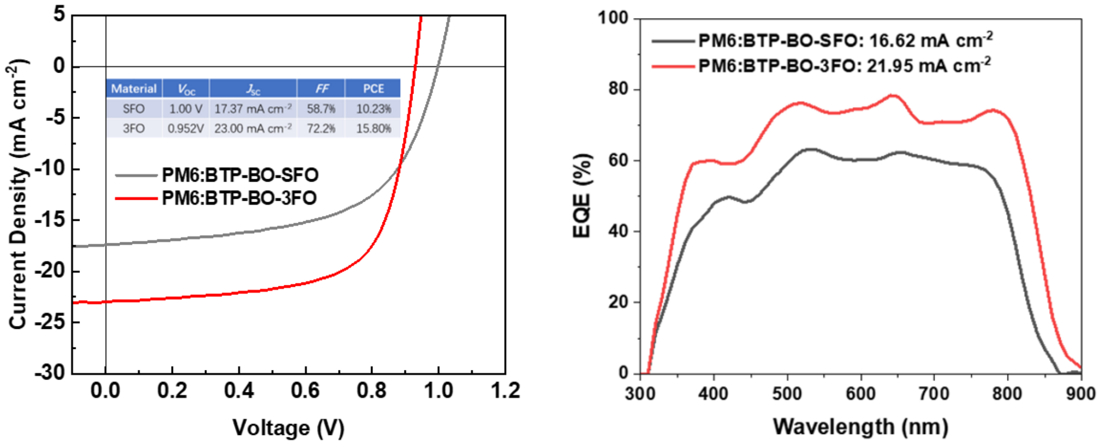

Fig. S12 *J-V* curves of binary BTP-BO-SFO and BTP-BO-3FO devices, and their EQE spectra

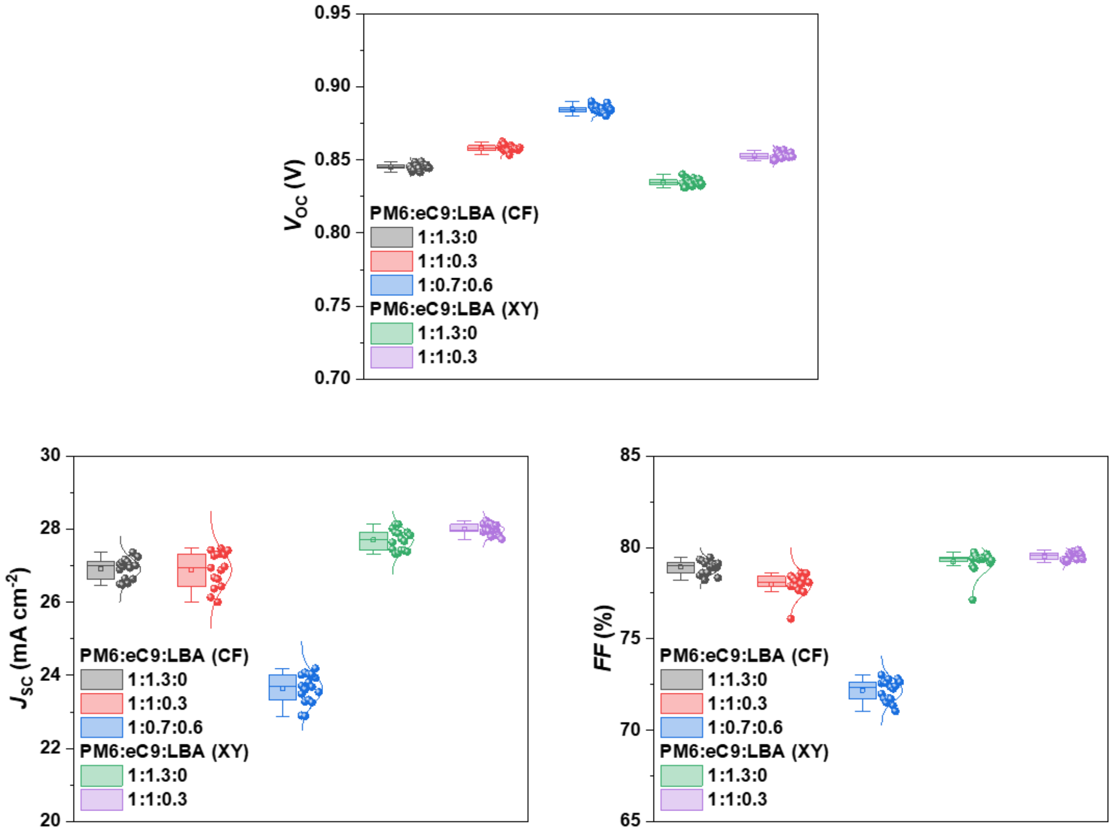

Fig. S13 Normal distribution of V_{OC}, J_{SC}, and *FF*, based on at least 10 devices

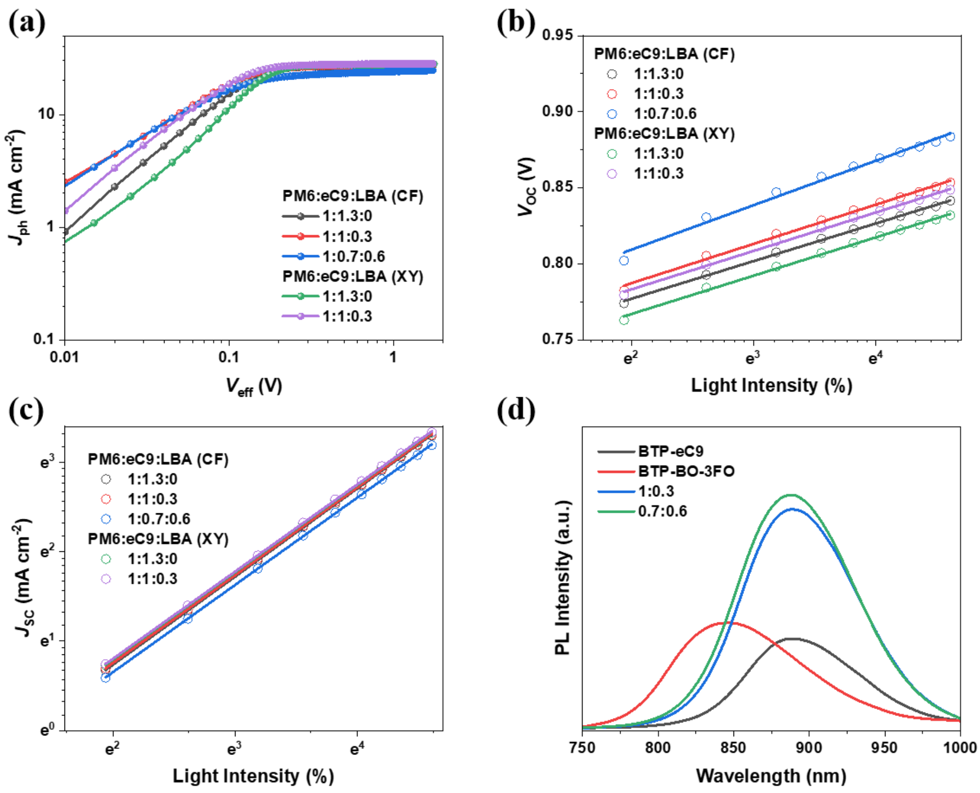

Fig. S14 (**a**) J_{ph} vs V_{eff} relationships. (**b**) light intensity vs V_{OC} and (**c**) vs J_{SC} curves. (**d**) PL spectra of BTP-eC9:BTP-BO-3FO films

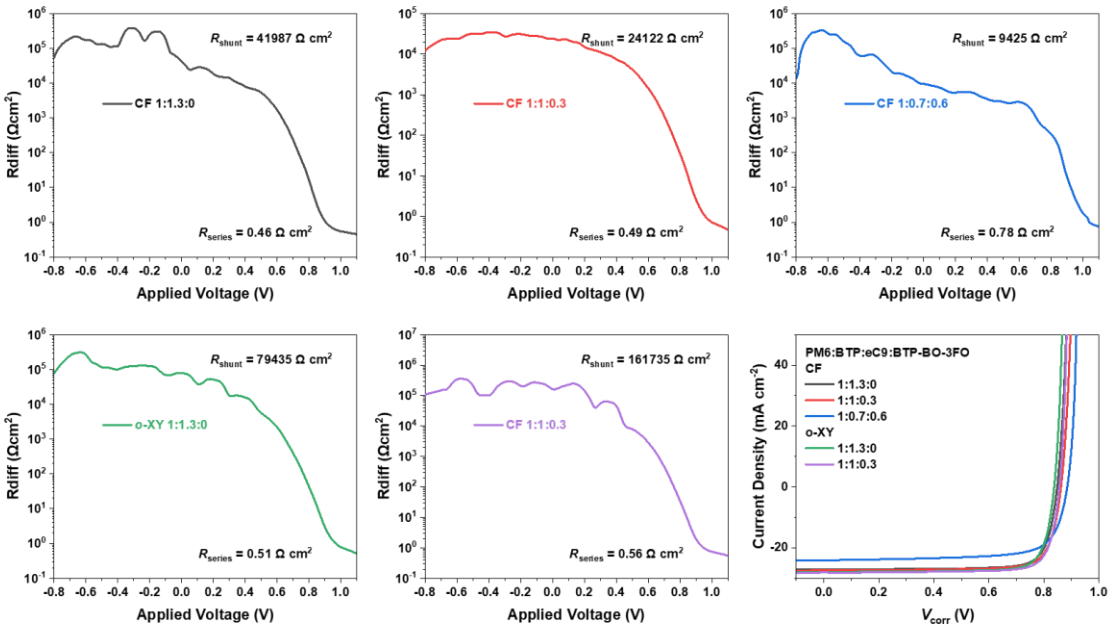

Fig. S15 Calculated series resistance and shunt resistance of all systems, and new J-V curves with corrected voltage

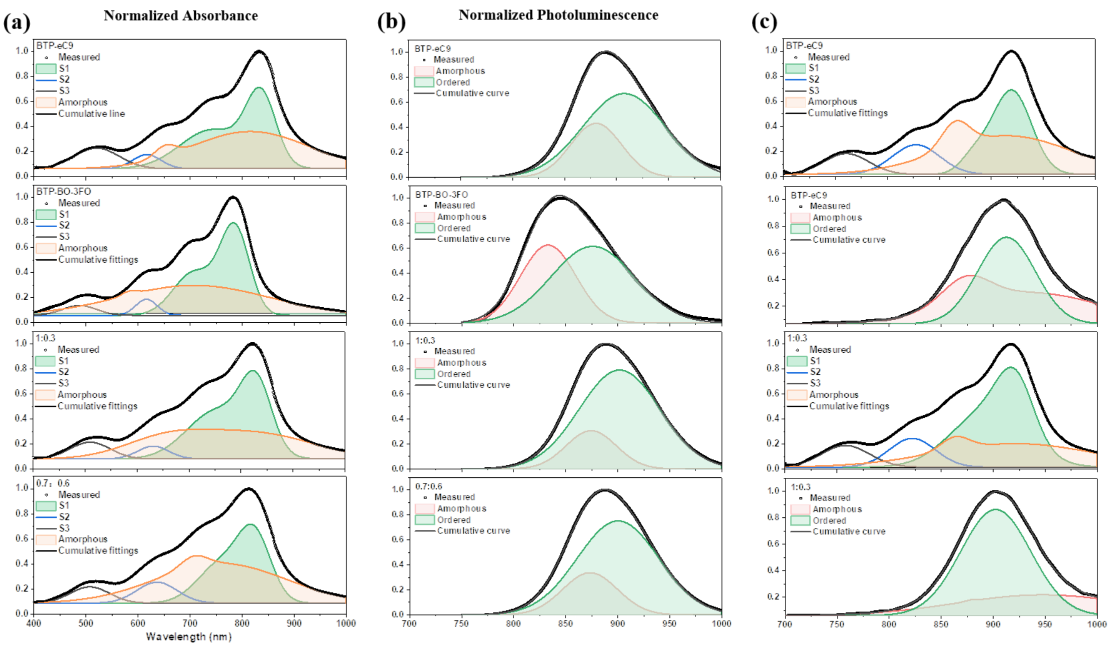

Fig. S16 CF processed acceptor-only films: (**a**) UV-vis spectra and (**b**) PL spectra. (**c**) UV-vis and PL spectra of *o*-XY processed ones. All are analyzed by gaussian fitting

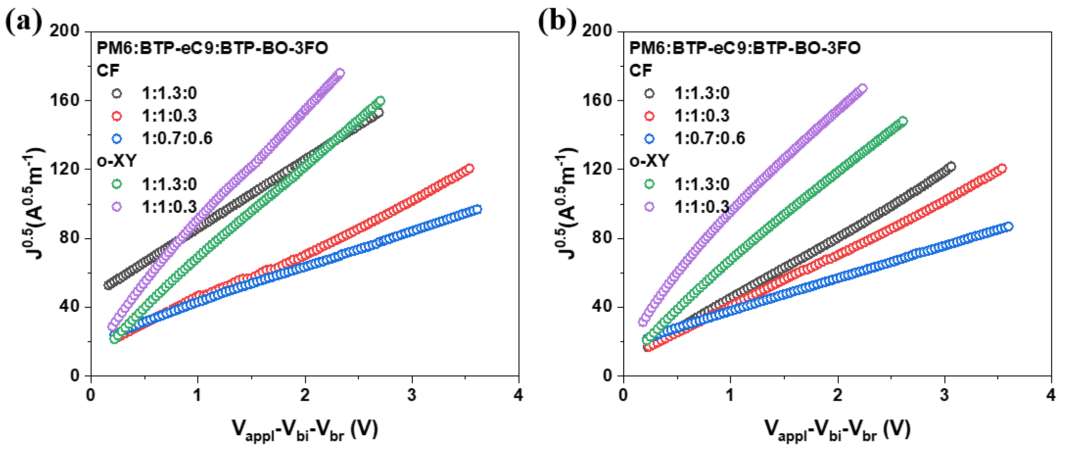

Fig. S17 (**a**) Hole-only and (**b**) electron-only device results

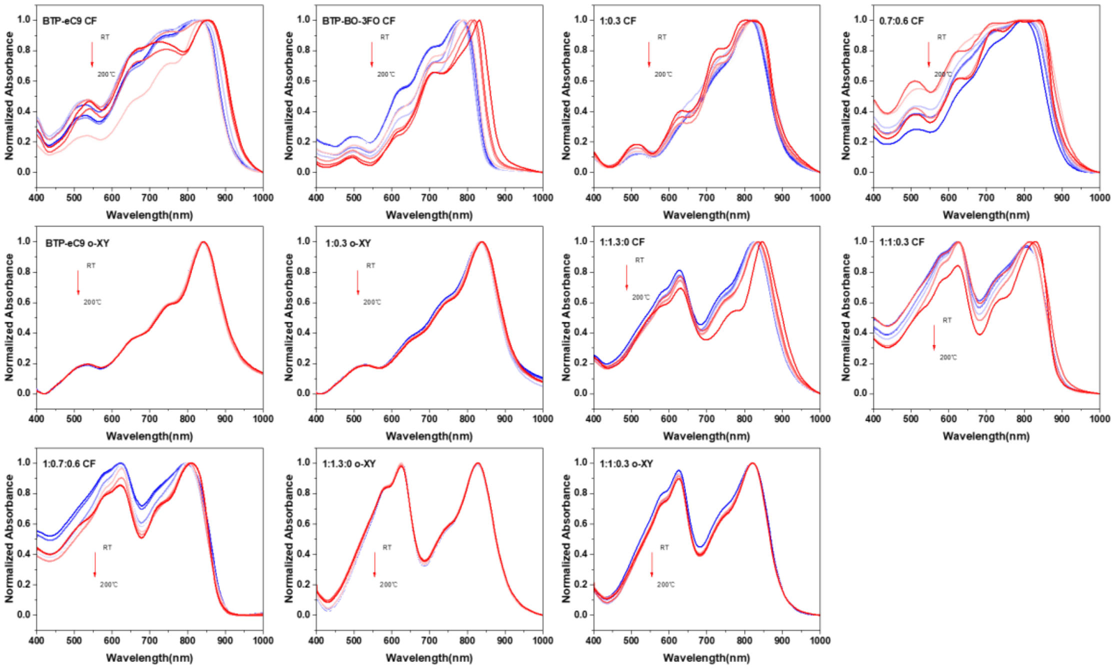

Fig. S18 Temperature varied thermally annealed film's absorption spectra

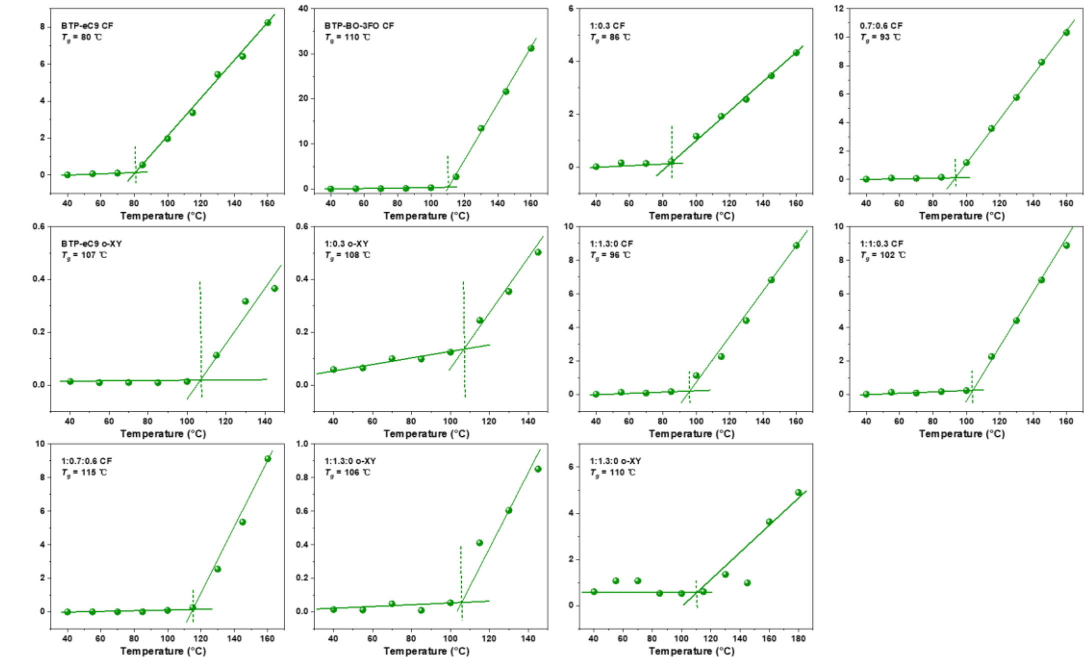

Fig. S19 UV-vis spectra deviation metrices of all neat and blend films

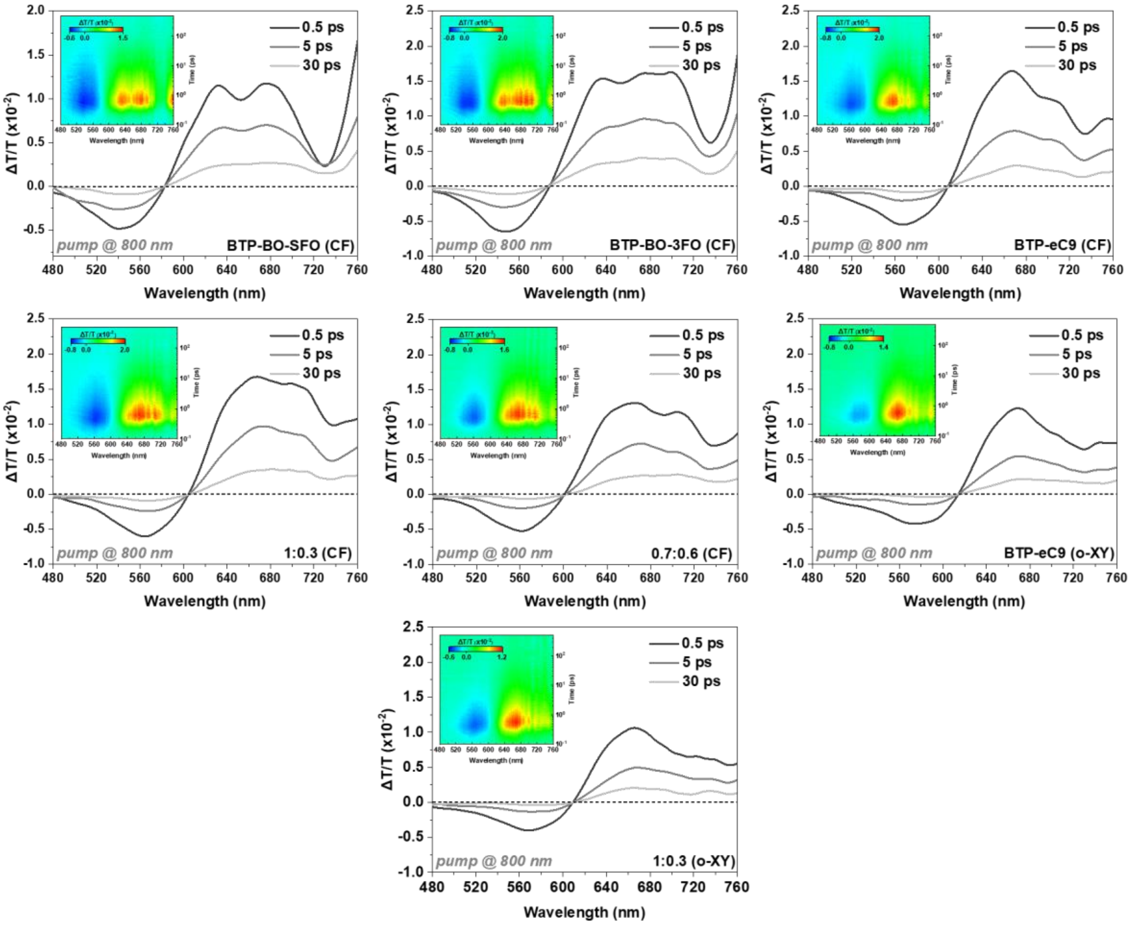

Fig. S20 TAS spectra with 2D color maps for acceptor-only films using 800 nm pump laser

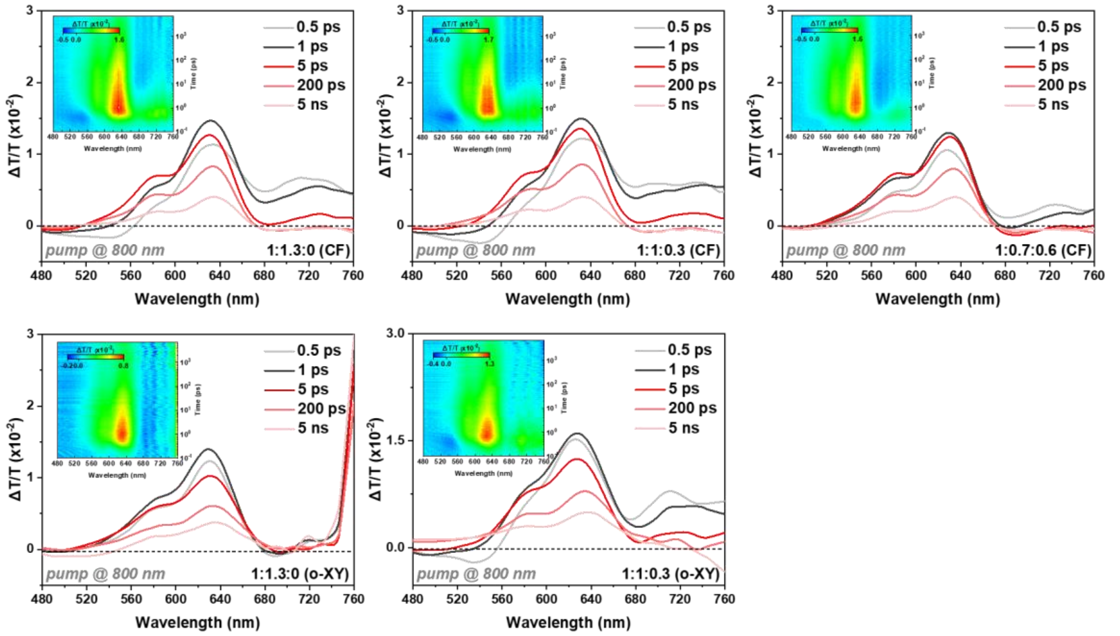

Fig. S21 TAS spectra with 2D color maps for blend films using 800 nm pump laser

Table S1 Photovoltaic performances of non-halogenated main solvent processed OSCs summary

Refs	V_{OC} (V)	J_{SC} (mA cm^{-2})	*FF* (%)	PCE (%)
**S1**	0.86	26.33	0.77	17.43
**S2**	0.851	26.75	0.8	18.25
**S3**	0.853	27.25	0.7814	18.16
**S4**	0.879	26.7	0.809	19
**S5**	0.85	25.76	0.781	17.12
**S6**	0.933	22.52	0.738	15.51
**S7**	0.947	22.78	0.746	16.1
**S8**	0.85	25.2	0.75	16.1
**S9**	0.84	26.9	0.796	18
**S10**	0.91	20.5	0.74	13.8
**S11**	0.94	19	0.7	12.5
**S12**	0.865	26.05	0.77	17.36
**S13**	0.856	24.94	0.755	16.1
**S14**	0.84	26.23	0.75	16.52
**S15**	0.96	17.97	0.7	12.1
**S16**	0.95	18.19	0.7	12.22
**S17**	1	18.9	0.63	11.9
**S18**	0.92	22.47	0.667	13.8
**S19**	0.88	17.62	0.76	11.76
**S20**	0.784	19.8	0.73	11.7
**S21**	0.85	26.1	0.78	17.33
**S22**	0.95	22.1	0.741	15.62
**S23**	0.88	24.3	0.726	15.6
**S24**	0.89	21.1	0.76	14.2
**S25**	0.89	23.4	0.67	13.97
**S26**	0.97	18.74	0.72	13.1
**S27**	0.95	18.67	0.71	12.6
**S28**	0.83	19.2	0.74	11.83
**S29**	0.78	20.37	0.73	11.77
**S30**	1.01	17.89	0.63	11.39

**S31**	0.82	28.15	0.778	18
**S32**	0.85	26.2	0.789	17.6
**S33**	0.856	26.535	0.793	18.02
**S34**	0.876	27.11	0.7641	18.14
**S35**	0.855	26.34	0.762	17.16
**S36**	0.920	24.3	0.807	18.0
**S37**	0.874	27.12	0.8026	19.07
**S38**	0.855	27.85	0.801	19.10
**This work**	0.857	28.13	79.8	19.24

Table S2 J_{ph} vs V_{eff} relationship derived parameters

**Systems**	J_{sat} **(mA cm^{-2})**	J_{SC} **(mA cm^{-2})**	J_{MPP} **(mA cm^{-2})**	η_{diss}, η_{coll} **(%)**
CF				
1:1.3:0	27.38	27.17	25.06	99.2, 91.5
1:1:0.3	27.82	27.42	25.12	98.5, 90.3
1:0.7:0.6	24.73	24.07	20.83	97.3, 84.2
o-XY				
1:1.3:0	28.24	27.97	25.89	99.0, 91.7
1:1:0.3	28.35	28.13	25.98	99.2, 91.6

The brackets contain averages and standard errors of PCEs based on 20 devices.

Table S3 Calculated parameters for (010) peak from OOP direction

**Systems**	Peak position (Å^{-1})	d-spacing (Å)	CCL (Å)
**CF**			
1:1.3:0	1.71; 1.80	3.68; 3.48	42.0; 57.6
1:1:0.3	1.78	3.55	22.0
1:0.7:0.6	1.76	3.58	7.62
**XY**			
1:1.3:0	1.72	3.65	45.8
1:1:0.3	1.68	3.73	33.6

Table S4 Mobilities

Systems	μ_h (10^{-4} cm^2V^{-1} s^{-1})	μ_e (10^{-4} cm^2V^{-1} s^{-1})	μ_h/μ_e
CF			
1:1.3:0	5.3	4.9	1.08
1:1:0.3	3.8	3.2	1.15
1:0.7:0.6	1.4	1.2	1.67
o-XY			
1:1.3:0	9.7	9.1	1.07
1:1:0.3	15	14	1.07

Supplementary References

[S1] D. Wang, H. Liu, Y. Li, G. Zhou, L. Zhan et al., High-performance and eco-friendly semitransparent organic solar cells for greenhouse applications. Joule **5**(4), 945-957 (2021). https://doi.org/https://doi.org/10.1016/j.joule.2021.02.010

[S2] D. Wang, G. Zhou, Y. Li, K. Yan, L. Zhan et al., High-performance organic solar cells from non-halogenated solvents. Adv. Funct. Mater. **32**(4), 2107827 (2022). https://doi.org/https://doi.org/10.1002/adfm.202107827

[S3] R. Sun, T. Wang, Y. Wu, M. Zhang, Y. Ma et al., Pedot:Pss-free polymer non-fullerene polymer solar cells with efficiency up to 18.60% employing a binary-solvent-chlorinated ito anode. Adv. Funct. Mater. **31**(51), 2106846 (2021). https://doi.org/https://doi.org/10.1002/adfm.202106846

[S4] Y. Cui, Y. Xu, H. Yao, P. Bi, L. Hong et al., Single-junction organic photovoltaic cell with 19% efficiency. Adv. Mater. **33**(41), 2102420 (2021). https://doi.org/https://doi.org/10.1002/adma.202102420

[S5] H. Chen, H. Lai, Z. Chen, Y. Zhu, H. Wang et al., 17.1 %-efficient eco-compatible organic solar cells from a dissymmetric 3d network acceptor. Angew. Chem. Int. Ed. **60**(6), 3238-3246 (2021). https://doi.org/https://doi.org/10.1002/anie.202013053

[S6] L. Jin, R. Ma, H. Liu, W. Xu, Z. Luo et al., Boosting highly efficient hydrocarbon solvent-processed all-polymer-based organic solar cells by modulating thin-film morphology. ACS Appl. Mater. Interfaces. **13**(29), 34301-34307 (2021). https://doi.org/10.1021/acsami.1c07946

[S7] S. Ding, R. Ma, T. Yang, G. Zhang, J. Yin et al., Boosting the efficiency of

non-fullerene organic solar cells via a simple cathode modification method. ACS Appl. Mater. Interfaces. **13**(43), 51078-51085 (2021). https://doi.org/10.1021/acsami.1c16550

[S8] L. Hong, H. Yao, Z. Wu, Y. Cui, T. Zhang et al., Eco-compatible solvent-processed organic photovoltaic cells with over 16% efficiency. Adv. Mater. **31**(39), 1903441 (2019). https://doi.org/https://doi.org/10.1002/adma.201903441

[S9] Y. Xu, Y. Cui, H. Yao, T. Zhang, J. Zhang et al., A new conjugated polymer that enables the integration of photovoltaic and light-emitting functions in one device. Adv. Mater. **33**(22), 2101090 (2021). https://doi.org/https://doi.org/10.1002/adma.202101090

[S10] C. Y. Liao, Y. Chen, C. C. Lee, G. Wang, N. W. Teng et al., Processing strategies for an organic photovoltaic module with over 10% efficiency. Joule **4**(1), 189-206 (2020). https://doi.org/https://doi.org/10.1016/j.joule.2019.11.006

[S11] H. Guo, Y. Zhang, L. Chen, X. Liao, Q. Xie et al., Non-halogenated-solvent-processed highly efficient organic solar cells with a record open circuit voltage enabled by noncovalently locked novel polymer donors. J. Mater. Chem. A **7**(48), 27394-27402 (2019). https://doi.org/10.1039/C9TA10624C

[S12] R. Ma, T. Yang, Y. Xiao, T. Liu, G. Zhang et al., Air-processed efficient organic solar cells from aromatic hydrocarbon solvent without solvent additive or post-treatment: Insights into solvent effect on morphology. Energy Environ. Sci. **5**(3), 977-985 (2022). https://doi.org/https://doi.org/10.1002/eem2.12226

[S13] S. Dong, T. Jia, K. Zhang, J. Jing, F. Huang. Single-component non-halogen solvent-processed high-performance organic solar cell module with efficiency over 14%. Joule **4**(9), 2004-2016 (2020). https://doi.org/https://doi.org/10.1016/j.joule.2020.07.028

[S14] R. Sun, T. Wang, Z. Luo, Z. Hu, F. Huang et al., Achieving eco-compatible organic solar cells with efficiency >16.5% based on an iridium complex-incorporated polymer donor. Sol. RRL **4**(7), 2000156 (2020). https://doi.org/10.1002/solr.202000156

[S15] Y. Qin, L. Ye, S. Zhang, J. Zhu, B. Yang et al., A polymer design strategy toward green solvent processed efficient non-fullerene polymer solar cells. J. Mater. Chem. A **6**(10), 4324-4330 (2018). https://doi.org/10.1039/C8TA00368H

[S16] Y. Xiong, L. Ye, A. Gadisa, Q. Zhang, J. J. Rech, W. You, H. Ade. Revealing the impact of f4-tcnq as additive on morphology and performance of high-efficiency nonfullerene organic solar cells. Adv. Funct. Mater. **29**(1), 1806262 (2019). https://doi.org/https://doi.org/10.1002/adfm.201806262

[S17] D. Liu, B. Yang, B. Jang, B. Xu, S. Zhang et al., Molecular design of a wide-band-gap conjugated polymer for efficient fullerene-free polymer solar cells. Energy Environ. Sci. **10**(2), 546-551 (2017). https://doi.org/10.1039/C6EE03489F

[S18] C. Zhu, Z. Li, W. Zhong, F. Peng, Z. Zeng et al., Constructing a new polymer acceptor enabled non-halogenated solvent-processed all-polymer solar cell with an efficiency of 13.8%. Chem. Commun. **57**(7), 935-938 (2021). https://doi.org/10.1039/D0CC07213C

[S19] L. Zhu, W. Zhong, C. Qiu, B. Lyu, Z. Zhou et al., Aggregation-induced multilength scaled morphology enabling 11.76% efficiency in all-polymer solar cells using printing fabrication. Adv. Mater. **31**(41), 1902899 (2019). https://doi.org/10.1002/adma.201902899

[S20] J. B. Zhao, Y. K. Li, G. F. Yang, K. Jiang, H. R. Lin et al., Efficient organic solar cells processed from hydrocarbon solvents. Nat. Energy **1**, 15027 (2016). https://doi.org/10.1038/nenergy.2015.27

[S21] X. Xu, L. Yu, H. Yan, R. Li, Q. Peng. Highly efficient non-fullerene organic solar cells enabled by a delayed processing method using a non-halogenated solvent. Energy Environ. Sci. **13**(11), 4381-4388 (2020). https://doi.org/10.1039/D0EE02034F

[S22] B. Liu, H. Sun, J.-W. Lee, J. Yang, J. Wang et al., Achieving highly efficient all-polymer solar cells by green-solvent-processing under ambient atmosphere. Energy Environ. Sci. **14**(8), 4499-4507 (2021). https://doi.org/10.1039/D1EE01310F

[S23] B. Du, Y. Ma, C. Guo, J. Cai, D. Li et al., Hot-casting boosts efficiency of halogen-free solvent processed non-fullerene organic solar cells. Adv. Funct. Mater. **31**(45), 2105794 (2021). https://doi.org/https://doi.org/10.1002/adfm.202105794

[S24] Y. Cui, H. Yao, L. Hong, T. Zhang, Y. Xu et al., Achieving over 15% efficiency in organic photovoltaic cells via copolymer design. Adv. Mater. **31**(14), 1808356 (2019). https://doi.org/https://doi.org/10.1002/adma.201808356

[S25] S. J. Jeon, Y. W. Han, D. K. Moon. 13.9%-efficiency and eco-friendly nonfullerene polymer solar cells obtained by balancing molecular weight and solubility in chlorinated thiophene-based polymer backbones. Small **15**(41), 1902598 (2019). https://doi.org/https://doi.org/10.1002/smll.201902598

[S26] W. Zhao, S. Zhang, Y. Zhang, S. Li, X. Liu et al., Environmentally friendly solvent-processed organic solar cells that are highly efficient and adaptable for the blade-coating method. Adv. Mater. **30**(4), 1704837 (2018). https://doi.org/https://doi.org/10.1002/adma.201704837

[S27] X. Xu, T. Yu, Z. Bi, W. Ma, Y. Li., Realizing over 13% efficiency in green-solvent-processed nonfullerene organic solar cells enabled by 1,3,4-thiadiazole-based wide-bandgap copolymers. Adv. Mater. **30**(3), 1703973 (2018). https://doi.org/https://doi.org/10.1002/adma.201703973

[S28] T. Kumari, S. M. Lee, S. H. Kang, S. Chen, C. Yang. Ternary solar cells with a mixed face-on and edge-on orientation enable an unprecedented efficiency of 12.1%. Energy Environ. Sci. **10**(1), 258-265 (2017). https://doi.org/10.1039/c6ee02851a

[S29] S. Rasool, Q. V. Hoang, D. Van Vu, T. T. Trang Bui, S.-M. Jin et al., High-efficiency non-halogenated solvent processable polymer/pcbm solar cells via fluorination-enabled optimized nanoscale morphology. J. Mater. Chem. A **7**(43), 24992-25002 (2019). https://doi.org/10.1039/C9TA08960H

[S30] Y. Tang, J. Yu, H. Sun, Z. Wu, C. W. Koh et al., Two compatible polymer donors enabling ternary organic solar cells with a small nonradiative energy loss and broad composition tolerance. Sol. RRL **4**(11), 2000396 (2020). https://doi.org/https://doi.org/10.1002/solr.202000396

[S31] B. Fan, F. Lin, J. Oh, H. Fu, W. Gao et al., Enabling high efficiency of hydrocarbon-solvent processed organic solar cells through balanced charge generation and non-radiative loss. Adv. Energy Mater. **11**(41), 2101768 (2021). https://doi.org/https://doi.org/10.1002/aenm.202101768

[S32] X. Song, P. Sun, D. Sun, Y. Xu, Y. Liu et al., Investigation of tunable halogen-free solvent engineering on aggregation and miscibility towards high-performance organic solar cells. Nano Energy **91**, 106678 (2022). https://doi.org/https://doi.org/10.1016/j.nanoen.2021.106678

[S33] R. Ma, C. Yan, P. W. K. Fong, J. Yu, H. Liu et al., In situ and ex situ investigations on ternary strategy and co-solvent effects towards high-efficiency organic solar cells. Energy Environ. Sci. **15**(6), 2479-2488 (2022). https://doi.org/10.1039/D2EE00740A

[S34] H. Lu, H. Wang, G. Ran, S. Li, J. Zhang et al., Random terpolymer enabling high-efficiency organic solar cells processed by nonhalogenated solvent with a low nonradiative energy loss. Adv. Funct. Mater. **32**(34), 2203193 (2022). https://doi.org/https://doi.org/10.1002/adfm.202203193

[S35] Y. Li, J. Wu, H. Tang, X. Yi, Z. Liu et al., Non-halogenated solvents and layer-by-layer blade-coated ternary organic solar cells via cascade acceptor adjusting morphology and crystallization to reduce energy loss. ACS Appl. Mater. Interfaces **14**(27), 31054-31065 (2022). https://doi.org/10.1021/acsami.2c05504

[S36] J. Wang, Y. Cui, Y. Xu, K. Xian, P. Bi et al., A new polymer donor enables binary all-polymer organic photovoltaic cells with 18% efficiency and excellent mechanical robustness. Adv. Mater. **34**(35), 2205009 (2022). https://doi.org/https://doi.org/10.1002/adma.202205009

[S37] L. Zhan, S. Yin, Y. Li, S. Li, T. Chen et al., Multiphase morphology with enhanced carrier lifetime via quaternary strategy enables high-efficiency, thick-film, and large-area organic photovoltaics. Adv. Mater. **34**(45), 2206269 (2022). https://doi.org/https://doi.org/10.1002/adma.202206269

[S38] R. Ma, X. Jiang, J. Fu, T. Zhu, C. Yan et al., Revealing the underlying solvent effect on film morphology in high-efficiency organic solar cells through combined ex situ and in situ observations. Energy Environ. Sci. **16**(5), 2316-2326 (2023). https://doi.org/10.1039/D3EE00294B
